# Plastid phylogenomics of tribe Perseeae (Lauraceae) yields insights into the evolution of East Asian subtropical evergreen broad-leaved forests

**DOI:** 10.1186/s12870-021-03413-8

**Published:** 2022-01-13

**Authors:** Tian-Wen Xiao, Hai-Fei Yan, Xue-Jun Ge

**Affiliations:** 1 Key Laboratory of Plant Resources Conservation and Sustainable Utilization, South China Botanical Garden, Chinese Academy of Sciences, Guangzhou, China; 2 University of Chinese Academy of Sciences, Beijing, China; 3 Center of Conservation Biology, Core Botanical Gardens, Chinese Academy of Sciences, Guangzhou, China

**Keywords:** Tribe Perseeae, EBLFs, Diversification rates

## Abstract

**Background:**

The East Asian subtropical evergreen broad-leaved forests (EBLFs) harbor remarkable biodiversity. However, their historical assembly remains unclear. To gain new insights into the assembly of this biome, we generated a molecular phylogeny of one of its essential plant groups, the tribe Perseeae (Lauraceae).

**Results:**

Our plastid tree topologies were robust to analyses based on different plastid regions and different strategies for data partitioning, nucleotide substitution saturation, and gap handling. We found that tribe Perseeae comprised six major clades and began to colonize the subtropical EBLFs of East Asia in the early Miocene. The diversification rates of tribe Perseeae accelerated twice in the late Miocene.

**Conclusions:**

Our findings suggest that the intensified precipitation in East Asia in the early Miocene may have facilitated range expansions of the subtropical EBLFs and establishment of tribe Perseeae within this biome. By the late Miocene, species assembly and diversification within the EBLFs had become rapid.

**Supplementary Information:**

The online version contains supplementary material available at 10.1186/s12870-021-03413-8.

## Background

Subtropical evergreen broad-leaved forests (EBLFs) are an important vegetation type, and are primarily distributed in East Asia [1]. EBLFs are characterized by species that depend on the regional monsoons, including *Castanopsis* Spach, *Lithocarpus* Bl., *Quercus* L. sect. *Cyclobalanopsis* Schneid. (Fagaceae), *Machilus* Nees (Lauraceae), and *Schima* Reinw. (Theaceae) [2]. Subtropical EBLFs presently occur between 24°N and 32°N and 99°E and 123°E in China, and they once covered about 25% of the total area of the country [3]. Unfortunately, over the past decades, the subtropical EBLFs have become dramatically reduced because of human activities [4].

Biomes, such as the EBLFs of East Asia, are assembled via a complex network of processes that occur at many spatial and temporal scales [5]. Assembly is accomplished from a regional species pool and is constrained by the unique biology of the species, their ecological tolerances, and their evolutionary histories [6]. Thus, assembly of a biome may be best understood through integrating over local, short-term ecological processes and longer-term, broader-scale evolutionary ones. Achieving a better understanding of the assembly of a biome and its historical dynamics can yield insights into the future of its biodiversity in a changing world [7, 8]. However, our knowledge regarding the historical assembly of the East Asian subtropical EBLFs remains limited.

Assembly and historical dynamics of regional biomes can be inferred by reconstructing ancestral states across dated phylogenies. In the past decades, several approaches to performing such analyses have been proposed (e.g., [9–11]). These approaches have been widely used to investigate the ancestral state of taxa and the evolution of biotas. For example, De-Nova et al. [12] employed a maximum likelihood approach to infer that seasonally dry tropical forest was the ancestral habitat of *Bursera* Jacq. (Burseraceae). The subtropical EBLFs of East Asia have also been investigated using ancestral state reconstructions (ASRs) of plant lineages. For example, Yu et al. [13] reconstructed the ancestral habitat of Theaceae and found that this family colonized the East Asian subtropical EBLFs in the early Miocene. Their results suggested that the evolution of the subtropical EBLFs of East Asia was facilitated by two intensifications of the East Asian monsoon (EAM), one at the Oligocene-Miocene (O-M) boundary and the other in the late Miocene [13]. In addition to ancestral range/state reconstructions, diversification rate analyses have also been used to explore the historical dynamics of the subtropical EBLFs of East Asia. For example, Yu et al. [13] and Wang et al. [14] detected accelerated diversification rates in Theaceae and Lardizabalaceae, respectively, in the Miocene.

Within the East Asian subtropical EBLFs, keystone families primarily comprise the Fagaceae, Lauraceae, Theaceae, and Magnoliaceae [15], all of which consist largely or entirely of tree species. Studies based on these keystone lineages may yield the most robust and accurate insights into the development of the EBLFs. Nevertheless, many prior studies employing historical biogeographic or diversification rate analyses have focused on herbs (*Coptis* Salisb.) [16], woody climbers, or shrubs (*Sabia* Colelbr. Lardizabalaceae) [14, 17]. Only two keystone plant groups of the East Asian subtropical EBLFs, Theaceae [13] and *Quercus* sect. *Cyclobalanopsis* (Fagaceae) [8], have been studied to investigate the historical assembly of this biome. The evolutionary histories of the other keystone plant groups within the EBLFs and their roles as components of biome assembly remain unclear.

In this study, we investigated historical assembly and change in the subtropical EBLFs of East Asia using the tribe Perseeae in the keystone family Lauraceae. Tribe Perseeae comprises eight genera and ca. 385 species (Table S1) that are shrubs and trees of great economic and ecological importance [18]. Species of tribe Perseeae are mainly distributed in tropical and subtropical Asia and belong to *Alseodaphne* Nees, *Alseodaphnopsis* H.W. Li & J. Li, *Apollonias* Nees, *Dehaasia* Bl., *Machilus* Nees, *Nothaphoebe* Bl., *Persea* Mill., and *Phoebe* Nees [19–21]. Among these genera, species of *Machilus* and *Phoebe* are particularly abundant in subtropical EBLFs of East Asia [15]. In *Machilus*, 40 of ca. 114 (35.1%) species are endemic to the East Asian subtropical EBLFs, and 12 of ca. 51 (23.5%) *Phoebe* species are endemic in this biome [18, 22]. An additional 33 species of *Machilus* (28.9%) occur more broadly within both the tropical forests and subtropical EBLFs of East Asia, and the same is true for 11 species of *Phoebe* (21.6%). Overall, the distributional pattern of tribe Perseeae and its abundance within the subtropical EBLFs of East Asia as a member of an ecologically critical family make it an ideal system for investigating the assembly and historical dynamics of this biome.

Although tribe Perseeae is an essential group within the East Asian subtropical EBLFs, its phylogenetic relationships are not fully resolved, partly due to hybridization among species and taxonomic difficulties [23, 24]. For example, a molecular phylogeny by Li et al. [21] involved considerable sampling at the species level, but the major clades of tribe Perseeae were not strongly supported based on the ITS and intron II of LEAFY. More recently, well-resolved phylogenies of tribe Perseeae were reconstructed using plastid genomes (plastomes), but *Machilus* and *Phoebe* were poorly sampled in these studies [25, 26].

In this study, we sampled 52 species of tribe Perseeae, especially within the genera *Machilus* and *Phoebe*, to reconstruct a robust phylogeny of the tribe based on plastomes. Our sampling covered all major clades present in previous studies [21, 25, 26] and the key distributional ranges in Asia and the Americas, as well as seven of eight genera (excluding *Apollonias*, which is monotypic and distributed in India). We then carried out molecular dating analyses, estimations of diversification rates, and ASR to understand the evolutionary dynamics of tribe Perseeae, especially within the subtropical EBLFs of East Asia. Based on these results, we used tribe Perseeae as a proxy to infer aspects of assembly and historical change in the East Asian subtropical EBLFs. Thus, our work provides an additional evolutionary perspective on the historical dynamics of EBLFs in subtropical Asia based on a plant lineage that is dominant within the biome.

## Results

### Plastome features

Forty-four complete plastomes were newly sequenced and assembled, with lengths ranging from 151,780 bp (*Sassafras tzumu* Hemsl.) to 152,920 bp (*Alseodaphnopsis sichourensis* H. W. Li & J. Li) and AT contents ranging from 60.8 to 60.9%. All plastomes shared a typical quadripartite structure (Fig. S1): a large single-copy region (LSC), a small single-copy region (SSC), and two copies of an inverted repeat region (IR). Each plastome contained 113 distinct genes, including 30 transfer RNAs (tRNAs), four ribosomal RNAs (rRNAs), and 79 protein-coding genes. Among the 113 genes, 18 contained introns.

We extracted 242 loci from 61 complete plastomes and aligned them prior to phylogenetic analyses. Among these loci, *ycf1*, *ycf2*, and *ndhF*-*rpl32* contained more variable sites and parsimony informative sites than other loci (Table S2). The 242 loci had a large range of AT contents, from 36.5% (*trnD*-*GUC*) to 90.5% (*rpl2*-*rpl23*), indicating an uneven distribution of AT content across different plastid regions.

### Phylogenomic analyses

**Fig. 1:**
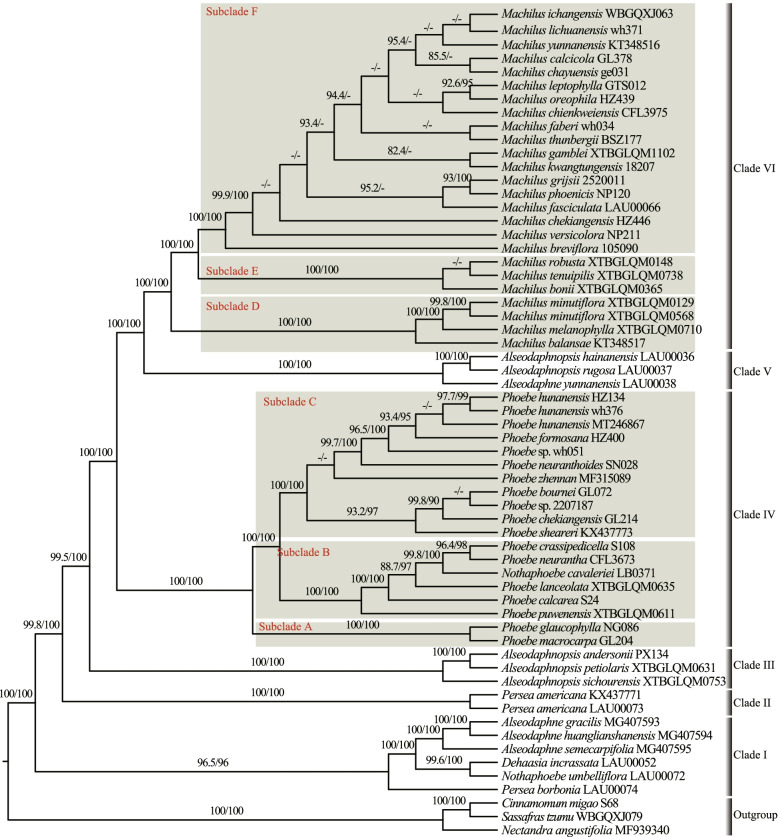
Maximum likelihood tree of tribe Perseeae based on unpartitioned complete plastomes (CP). The support values of Shimodaira-Hasegawa-like approximate likelihood ratio test (SH-aLRT ≥ 80%; on the left) and ultrafast bootstrap (UFBS ≥ 95%; on the right) are shown on branches. “-” above branches indicate SH-aLRT < 80% and UFBS < 95%, respectively

We generated 22 data matrices (Table 1) that differed in the types of plastid DNA data they contained, the approach used to find and remove potentially saturated loci, and the degree of gap removal. The alignment lengths, numbers of parsimony informative sites, AT contents, and best-fitting substitution models of all 22 matrices are shown in Table 1. We used the alignments to infer 22 maximum likelihood (ML) trees. The ML trees that resulted from an unpartitioned strategy were largely congruent with those inferred using a partitioned strategy (Figs. 1, and S2, S3, S4, S5, and S6). In the saturation analysis, IQ-TREE v1.6.12 [27] failed to build gene trees for 45 out of the 242 loci because of their lack of parsimony-informative sites (Table S2). Thus, only 197 loci and their gene trees were used to calculate patristic distance (PD) and *p* in TreSpEx v1.1 [28]. In the TreSpEx analysis, 55 loci exhibited possible saturation based on slope (Fig. S7a), and a slightly different set of 50 loci showed possible saturation based on R^2^ values (Fig. S7b). Consequently, we performed two independent ML analyses with the 55 (CP-slope) and 50 genes (CP-R^2^) removed, but we found that the resulting topologies (Figs. S8 and S9) were largely congruent with that based on complete plastomes (CP). Moreover, increasing the percentage of gaps in alignments did not affect tree topology (data not shown, but previously made available at Dryad [29]). The number of gaps among the alignments of 242 loci were highly variable, ranging from zero (e.g., *atpB*) to 28,413 (*ccsA*-*trnL*^*UAG*^) (Fig. S10).

**Table 1:** Summary of sequence matrices, tree inference strategies and best-fitting models in ML analyses. CP, complete plastomes; PCG, protein-coding genes; NPCG, non-protein coding genes; CP-slope, excluding saturated loci from CP based on slopes of the linear regression; CP-R^2^, excluding saturated loci from CP based on R^2^ of the linear regression; CP-gappy90, excluding sites with more than 90% gap percentage from CP; CP-gappy0, excluding all gap-containing sites from CP. Beat-fitting models of each locus under partitioned strategy for CP, PCG, and NPCG are not shown and indicated as “-”

Matrices	Alignment length (bp)	Parsimony informative sites	AT content of datasets (%)	Strategies	Models
CP / CP-p	141,881	1915	61.7	Unpartitioned / Partitioned	TVM+F+R5 / -
PCG / PCG-p	69,345	753	60.9	Unpartitioned / Partitioned	TVM+F+R2 / -
NPCG / NPCG-p	72,536	1162	62.4	Unpartitioned / Partitioned	K3Pu+F+R5 / -
CP-slope	99,898	1202	61.3	Unpartitioned	TVM+F+R2
CP-R^2^	101,321	1178	61.1	Unpartitioned	TVM+F+R2
CP-gappy90	138,627	1910	61.7	Unpartitioned	TVM+F+R5
CP-gappy70	138,282	1894	61.6	Unpartitioned	TVM+F+R5
CP-gappy50	138,133	1881	61.6	Unpartitioned	TVM+F+R5
CP-gappy25	137,808	1858	61.6	Unpartitioned	TVM+F+R4
CP-gappy15	137,738	1855	61.6	Unpartitioned	TVM+F+R4
CP-gappy13	137,726	1853	61.6	Unpartitioned	TVM+F+R4
CP-gappy12	137,716	1851	61.6	Unpartitioned	TVM+F+R4
CP-gappy10	135,865	1837	61.6	Unpartitioned	TVM+F+R4
CP-gappy09	131,254	1829	61.6	Unpartitioned	TVM+F+R4
CP-gappy07	130,998	1816	61.6	Unpartitioned	TVM+F+R4
CP-gappy05	130,948	1814	61.6	Unpartitioned	TVM+F+R4
CP-gappy04	130,658	1798	61.5	Unpartitioned	TVM+F+R4
CP-gappy02	127,918	1735	61.5	Unpartitioned	TVM+F+R4
CP-gappy0	112,034	1476	61.2	Unpartitioned	TVM+F+R3

All 22 ML tree topologies were largely congruent; we therefore present only the unpartitioned CP ML tree in the main text (Fig. 1), and we regard this tree as the species tree. All trees revealed that tribe Perseeae is a monophyletic group comprised of six strongly supported major clades based on Shimodaira-Hasegawa-like approximate likelihood ratio tests (SH-aLRTs) and ultrafast bootstrap (UFBS) (i.e., SH-aLRT = 95.3–100%, UFBS = 97–100%; Fig. 1). In clade I, *Alseodaphne gracilis* Kosterm., *A. huanglianshanensis* H.W. Li & Y.M. Shui, and *A. semecarpifolia* Nees formed a monophyletic group that was sister to *Dehaasia incrassata* Kosterm. and *Nothaphoebe umbelliflora* Bl., and *Persea borbonia* Spreng., a species distributed in the southeastern United States, was sister to the rest of the clade. Clade II contained *Persea americana* Mill., which is native to Central America, and its unique phylogenetic position indicates that *Persea* Mill. is not monophyletic. Clade III comprised three species of *Alseodaphnopsis* H.W. Li & J. Li: *A. andersonii* H.W. Li & J. Li, *A. petiolaris* H.W. Li & J. Li, and *A. sichourensis* H.W. Li & J. Li. In clade IV, *Nothaphoebe cavaleriei* H. Lév. was nested within the genus *Phoebe*. Clade V consisted of *Alseodaphnopsis hainanensis* H.W. Li & J. Li, *A. rugosa* H.W. Li & J. Li, and *Alseodaphne yunnanensis* Kosterm. Species of *Machilus* formed clade VI, which was sister to clade V.

Although the 22 ML trees were largely in agreement, the phylogenetic positions of several species of *Machilus* were unstable and showed low support (i.e., SH-aLRT < 80% or UFBS < 95%; Figs. 1, S2, S3, S4, S5, S6, S8 and S9).

### Divergence time estimation

Based on the application of criteria for clock-likeness, reasonable gene tree lengths, and concordance with the species tree, the genes *ycf1*, *ycf2* and *ndhF* were selected for molecular dating analyses. In an analysis in which we calibrated the stem node of clade IV (node 3, scenario 5; Table S3) using the fossil *Machilus maomingensis* Jin & Tang, we estimated the stem age of tribe Perseeae to be 57.69 million years old (Ma) (95% highest posterior density (HPD) = 46.96–68.39 Ma). Similarly, when we calibrated the stem node of clade III (node 4, scenario 3; Table S3) using the fossil *Alseodaphne changchangensis* Jin & Li, the stem age of tribe Perseeae was inferred to be 54.80 Ma (95% HPD = 46.0–64.57 Ma). These two independent dating analyses both yielded stem ages for tribe Perseeae close to 55.3 Ma, which we regard as an external standard based on a prior study (see Methods). Therefore, we used both *M. maomingensis* and *A. changchangensis* in a final dating analysis (nodes 3 and 4, scenario 6; Table S3).

Our final dating analysis in BEAST v2.6.3 [30] indicated that tribe Perseeae originated in the very late Paleocene (56.50 Ma; 95% HPD = 45.87–66.57 Ma; Fig. S11). The stem and crown age of *Phoebe* were estimated to be 35.82 Ma (95% HPD = 34.09–38.06 Ma) and 25.86 Ma (95% HPD = 16.73–34.77 Ma), respectively. The stem and crown age of *Machilus* were 26.33 Ma (95% HPD = 16.93–34.23 Ma) and 15.38 Ma (95% HPD = 8.65–23.9 Ma), respectively.

### Diversification rate and shift

**Fig. 2:**
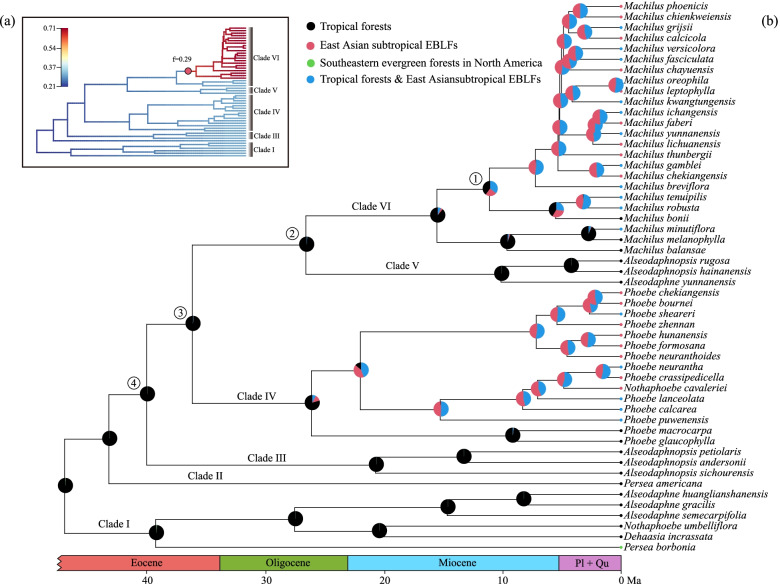
Combined diversification rate analyses and ancestral habitat reconstruction of tribe Perseeae. (**a**) Phylorate inferred from BAMM analysis with “ExpectedNumberofShifts” set to 1 and global sampling probability set to 0.135. (**b**) Ancestral habitat reconstructed by R package phytools with SYM model. Speciation rates (lineages per million years) are color-coded on branches in (**a**). Red circles in (**a**) indicates the position of speciation rate shift. Numbers in the circles near the nodes in (**b**) correspond to those node numbers listed in the main text and Fig. S11. The pie charts at the nodes represent the posterior probabilities of ancestral habitat types. Dated phylogeny of tribe Perseeae is derived from Fig. S11. Pl and Qu are abbreviations for Pliocene and Quaternary, respectively

**Fig. 3:**
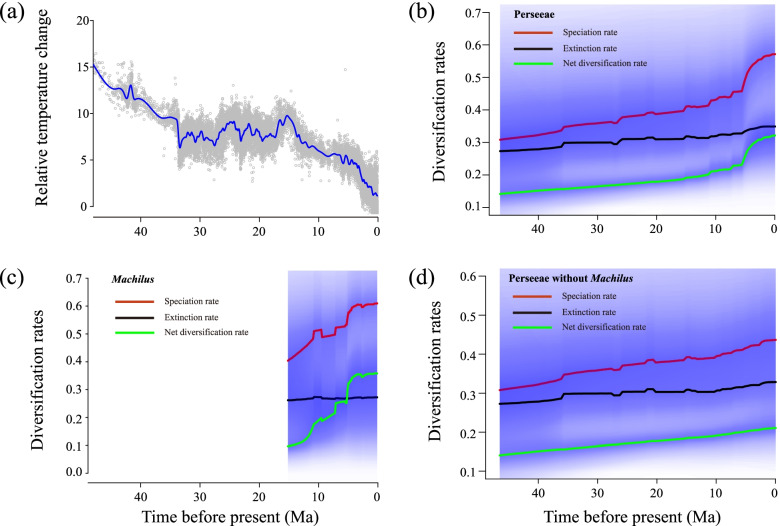
Speciation, extinction, and net diversification rates through time plots obtained from the BAMM analyses. (**a**) The global temperature change in the past 45 Ma is derived from Zachos et al. [113]. Plots (**b**), (**c**) and (**d**) are diversification rates for Perseeae, *Machilus*, and Perseeae without *Machilus*, respectively; speciation, extinction, and net diversification rates are colored in red, black, and green, respectively; the shaded areas denote 95% confidence intervals in rate reconstructions

Speciation rates obtained from Bayesian analysis of macro-evolutionary mixtures (BAMM) v2.5 [31] indicated diversification rate heterogeneity within tribe Perseeae (Fig. 2a). One diversification rate shift was detected within clade VI (near node 1, ca. 11 Ma; Fig. 2b) by sampling the maximum posterior configuration with the highest frequency (f = 0.29; Figs. 2a and S12). The rate-through-time plot suggested that the global speciation and net diversification rates of tribe Perseeae accelerated at ca. 11 Ma, followed by a steady increase from 11 Ma to 5.5 Ma and a rapid increase at ca. 5.5 Ma (Fig. 3b). Speciation and net diversification rates of *Machilus* increased rapidly, particularly at 11 Ma and 5.5 Ma (Fig. 3c). After excluding *Machilus*, speciation and net diversification rates of tribe Perseeae increased steadily and slowly from 46.48 Ma to the present (Fig. 3d). The diversification rate patterns and locations of rate shifts of tribe Perseeae were consistent under different global sampling probabilities (Figs. 2a and S13).

The speciation and extinction rates obtained using CPP on Mass-Extinction Times (CoMET) showed a pattern highly similar to that obtained using BAMM (Fig. S14). Specifically, the speciation rate accelerated at ca. 11 Ma and experienced a significant increase at 5.5 Ma (Fig. S14a). Moreover, we detected no mass extinction events. Within the CoMET analysis, the effective sample sizes (ESSs) of all parameters were greater than 200, and the Geweke statistic plots tended towards satisfactory, indicating reliability (Fig. S15).

### Ancestral habitat

Among the 52 sampled species of tribe Perseeae, 17 inhabit only tropical forests, 17 are endemic in the East Asian subtropical EBLFs, 17 are found in both tropical forests and the East Asian subtropical EBLFs, and one occurs in the southeastern evergreen forests of North America (Table S4). Thus, we coded the species according to their modern-day habitats within these three major vegetation types and reconstructed the ancestral habitat using stochastic character mapping (SIMMAP) [9]. In SIMMAP, we used the “symmetric” model (SYM), which was the best-fitting model according to the Akaike Information Criterion (AIC) (Table S5). Our analyses revealed that the most recent common ancestors (MRCAs) of *Phoebe* and *Machilus* occurred in tropical forests in the Oligocene and Miocene, respectively (Fig. 2b). *Phoebe* and *Machilus* began colonizing the East Asian subtropical EBLFs at ca. 21 Ma and 11 Ma, respectively (Fig. 2b).

## Discussion

### Phylogenetic relationships within tribe Perseeae

Previous studies have suggested that conflicting phylogenetic signals among different plastid regions and different strategies for data partitioning, and substitution saturation are all factors that can affect tree topologies [32–34]. Here, however, we found that the phylogenetic relationships of the six clades (clades I–VI) and the species relationships within clades I–V were robust to these factors across 22 ML tree reconstructions. This result strongly suggests that these factors had no or negligible influence on the tree topology of tribe Perseeae.

Gaps in sequences are thought to have especially strong effects on tree topologies and phylogenetic accuracy in some cases, although the exact nature of the relationship between gaps and phylogenetic inference remains somewhat unclear. For example, Duvall et al. [35] noted that alignment gaps often occur at sites of ambiguous poly-A/T sequences. Thus, these sequences, in particular, may affect phylogenetic inference. Consistent with Duvall et al. [35], we found that AT content decreased (from 61.7 to 61.4%) as gap content was reduced (from 90% gaps to 0% gaps) in our sequence matrices (Table 1). Aside from specific types of gaps, the distribution and complexity of gaps may also affect phylogenetic accuracy [36], and our data (CP) exhibited complex gap distributions (Fig. S10). However, topologies of 14 datasets representing different levels of gappy-ness (from CP-gappy90 to CP-gappy0; Table 1) all agreed with the topology of the CP ML tree, suggesting that neither ambiguous poly-A/T sequences nor the complex distribution of gaps influenced our tree topology.

Phylogenetic relationships of the six clades revealed by the CP tree (and other trees) were consistent with the coalescent-based plastome tree generated in ASTRAL III [37] by Liu et al. [26]. However, the ASTRAL plastome tree from that study and our CP tree were highly incongruent with the nuclear ribosomal DNA (nrDNA) ML tree of Liu et al. [26], which showed that clades III and V formed a strongly supported group (BS = 95%) that was sister to clade IV (BS = 79%). This differs from our result, in which clade VI is sister to clade V and clade III is sister to clades IV + V + VI. Significant incongruence between plastome and nrDNA phylogenies has been observed for many plant groups, such as *Heuchera* L. (Saxifragaceae) [38], the *Amelanchier*-*Malacomeles*-*Peraphyllum* clade (Rosaceae) [39], and Apioideae (Apiaceae) [40, 41]. Such incongruence may be attributed to sampling error, paralogy of nrDNA sequences (due to allopolyploidy or incomplete concerted evolution), incomplete lineage sorting (ILS), or hybridization and introgression [42, 43]. Similar to the nrDNA-ML tree of Liu et al. [26], previous phylogenetic studies using different species, accessions, and molecular markers (ITS + LEAFY intron II) revealed that clade V was closer to clade III rather than clade VI [19, 21]. Therefore, sampling error and paralogy of nrDNA may not cause incongruence in tribe Perseeae, and, notably, polyploids have not been found in the tribe to date [44]. Although ILS and hybridization/introgression can now be modeled using coalescent and network approaches, respectively, disentangling the two evolutionary phenomena remains challenging [45].

*Nothaphoebe cavaleriei* nested within *Phoebe* in our study, consistent with the results of Rohwer et al. [20]. Thus, we agree with the taxonomic treatment of *N. cavaleriei* within *Phoebe* [46]. In our study, *Phoebe* was divided into three subclades (subclades A, B, and C) with high support, but there were no obvious morphological synapomorphies uniting each clade. Morphological characters, such as the absence, presence, or type of hairs on outside tepals, have been traditionally used to delimit intrageneric sections within *Phoebe* [18]. However, the three subclades identified here are inconsistent with these morphologically defined sections. For example, *P. chekiangensis* C.B. Shang from sect. *Caniflorae* Meissn. was not grouped with *P. glaucophylla* H.W. Li and *P. macrocarpa* C.Y. Wu from the same section (Fig. 1).

In the genus *Machilus*, our phylogenetic results showed three strongly supported subclades (subclades D, E, and F). However, resolution within subclade F was poor, and this may be because of recent differentiation or decreased substitution rate in this subclade [20]. Thus, resolving the relationships within subclade F may require a large set of nuclear markers and approaches that utilize and visualize even weak signals in the data, such as simplex plots of tree concordance [47] and the *D*-statistic (i.e., “ABBA BABA”) [48].

### The robustness of diversification analysis with BAMM

Although incomplete sampling can potentially affect diversification rate analyses [49], BAMM has been shown to perform well under complete to low taxon sampling [50, 51]. To test the sensitivity of BAMM results to incorrect estimates of species diversities and incomplete sampling, Shi and Rabosky [52] ran an additional analysis with sampling probability halved and found that diversification rates were extremely similar under different sampling probabilities. We implemented a similar strategy and found that global sampling probability did not affect our results. Therefore, we have confidence in the results generated by BAMM. Nevertheless, our sampling of tribe Perseeae remains limited and it should be noted that biased taxon samplings (e.g., phylogenetic bias and spatial bias) can potentially lead to biasness in diversification rate patterns, which are inherent in large-scale biodiversity analyses [53–56].

### The expansion of subtropical EBLFs in the early Miocene

Communities and biomes respond to climate change through species dynamics [57]. Thus, the dynamics of Lauraceae, such as tribe Perseeae, may provide insights into the overall development of subtropical EBLFs because they are keystone species. Here, we found that tribe Perseeae did not colonize the subtropical EBLFs until the O-M boundary (21.77 Ma; Fig. 2), indicating that modern subtropical EBLFs of East Asia may not have originated until this time. Our result is congruent with the conclusion of Yu et al. [13] based on inferences from Theaceae, which also contains many keystone EBLF species. Other essential elements of the East Asian subtropical EBLFs, such as *Magnolia* L. sect. *Yulania* Dandy [58], also appear to have originated around the O-M boundary.

Lauraceae species were geographically widespread throughout the Late Cretaceous to early Eocene based on their common occurrence in fossil assemblages from North America, Europe, and Asia [59–61]. Fossils of tribe Perseeae and its sister groups (tribe Laureae and tribe Cinnamomeae) in the Eocene indicate that these tribes once colonized high latitudes of the Northern Hemisphere (e.g., the London Clay Flora [62], west and east Beringia [63]). Beringia was occupied by deciduous and evergreen broad-leaved forests (the boreotropical flora) in the Eocene [64], suggesting that tribe Perseeae colonized temperate or subtropical forests. As a response to dramatic climate change from the middle Eocene to the Oligocene, the boreotropical forests migrated to low latitude regions, and many temperate/subtropical lineages became extinct.

There was a broad arid/semi-arid belt between 20–40°N in East Asia from at least the Eocene [65]. The O-M boundary is broadly characterized by a brief glaciation in the Southern Hemisphere and global cooling [66], which, in eastern Asia, led to changes in moisture patterns [67] and in floral composition since the early Miocene [68]. The increase in humidity supported new, moist forests [69] and, thus, the immigration of plants from the lower-latitude paleotropics to new, higher-latitude, subtropical habitats [70]. It was not until the Neogene that the Lauraceae became common and dominant in the East Asian fossil flora [71, 72]. This is consistent with our ancestral habitat reconstruction results, which suggest that the MRCA of *Machilus* and *Phoebe* occurred in tropical forests (Fig. 2b) and that an ancestor of *Phoebe* colonized the East Asian subtropical EBLFs beginning at ca. 21 Ma, around the O-M boundary. The migration of *Machilus* into the EBLFs came later, around 11 Ma, and may be more consistent with a peak intensification of the EAM [73]. Overall, our results, if they can be applied broadly to the EBLFs, suggest that this biome underwent at least two major periods of diversification, first at the O-M boundary and again during a period of particularly strong regional monsoons.

### Rapid species accumulation of East Asian subtropical EBLFs in the late Miocene

Results from BAMM supported two periods of increased diversification rate within tribe Perseeae in the late Miocene (ca. 11 Ma and 5.5 Ma; Figs. 2a and 3). The two accelerations of diversification rate in the late Miocene were consistent with the CoMET analysis (Fig. S15). Thus, our results indicated that the late Miocene was pivotal for the assembly and evolution of tribe Perseeae and, by proxy, possibly for the modern subtropical EBLFs [14]. Notably, the acceleration in diversification ca. 5.5 Ma may coincide with a slight decrease in the intensity of the EAM [73], which may have opened up additional niches within the developing ELBFs.

High species diversity, such as that found in the EBLFs, may result from recent rapid speciation in a ‘cradle’ and/or the gradual accumulation and preservation of species over time in a ‘museum’. Some species within the EBLFs may be relicts from the early Cretaceous or late Jurassic period that survived in glacial refugia within hilly or mountainous regions of southern or eastern China where the EBLFs now occur [74]. However, unlike species of tribe Perseeae, most of these relicts are monotypic in subtropical EBLFs. By contrast, rapid species accumulation in the late Miocene has been reported for several dominant groups in subtropical EBLFs, such as *Schima* [13], *Camellia* L. (Theaceae) [13], and *Quercus* sect. *Cyclobalanopsis* [8].

Increased annual precipitation has sometimes been invoked to explain the higher diversification rates of plants and the rapid assembly of the East Asian subtropical EBLFs in the late Miocene (e.g., [13, 14, 75]). For example, Farnsworth et al. [76] indicated that a “super monsoon” existed from ca. 12 to ca. 4 Ma, resulting in increased annual precipitation. This is consistent with studies based on magnetic records and isotopic evidence that also support a progressively intensified EAM in the late Miocene and early Pliocene [77, 78]. Although the correlation between the intensified EAM and greater precipitation and increased diversification rates remains unclear, annual rainfall is the single best water-related predictor for species richness of Chinese woody plants [79]. Thus, the intensified rainfall associated with the EAM may have promoted periods of rapid assembly of the East Asian subtropical EBLFs in the late Miocene [13, 14, 75], consistent with our results for tribe Perseeae. However, fluctuations in the intensity of the monsoon may have opened additional niches through time and supported additional diversification.

## Conclusions

Our phylogenetic analyses have revealed six strongly-supported major clades within tribe Perseeae (Lauraceae) based on plastomes. *Machilus* and *Phoebe*, two dominant genera in the East Asian subtropical EBLFs, were monophyletic. Our results indicate that tribe Perseeae from tropical forests in the late Paleogene colonized the East Asian subtropical EBLFs in the early Miocene, and this was possibly facilitated by changes in global moisture patterns, including periods of change to the intensity of the EAM. In general, increases in moisture and humidity probably supported diversification in lineages of tribe Perseeae within the EBLFs. Along with prior results from Theaceae [13], which is also a dominant element of the ELBFs, our results suggest that the Miocene was a critical period for the assembly of the modern ELBFs. Moreover, our study illustrates how tribe Perseeae evolved under past climatic changes and, therefore, may provide insights into the possible responses of subtropical EBLFs to ongoing and future climate change.

## Methods

### Sampling, DNA sequencing, plastome assembly and annotation

We used a total of 43 samples for DNA sequencing in this study. These samples represented 37 species from four genera in tribe Perseeae and two species from tribe Cinnamomeae as an outgroup (Table S6). We used silica-gel-dried leaf tissues for DNA extraction. The first author identified and deposited voucher specimens in the herbarium of the South China Botanical Garden, Chinese Academy of Sciences (IBSC) (Table S6). We collected the plant materials from the wild and from botanical gardens. No specific permissions or licenses were required for our collections and experiments.

For DNA extraction, sequencing, plastome assembly, and annotation, we followed the methods of Xiao et al. [32]: we extracted genomic DNA using the cetyl trimethyl ammonium bromide (CTAB) method [80] and assembled and annotated plastomes with NOVOPlasty v2.7.2 [81] and GeSeq [82], respectively. We generated maps of annotated plastomes with the OrganellarGenomeDRAW tool (OGDRAW; [83]).

### Data preparation and phylogenetic analyses

In addition to newly sequenced plastomes, we also downloaded 17 plastomes representing 15 species of tribe Perseeae and one species of tribe Cinnamomeae (Table S7) from the National Center for Biotechnology Information (NCBI) [84] for use in phylogenetic analyses. Thus, our samples for phylogenetic reconstruction comprised 58 plastomes of tribe Perseeae, representing 53 species, and three plastomes of outgroup taxa, for a total of 61 plastid genomes. From all plastomes (newly sequenced and downloaded), we extracted protein-coding genes (PCGs), introns, intergenic spacers (IGSs), tRNAs, and rRNAs using a python script [85] and aligned the extracted loci separately using MAFFT v7.467 [86] in “localpair” mode with 1000 iterative refinements.

Inaccurate tree inference can arise for both statistical and biological reasons [87], including conflicting phylogenetic signals between coding and non-coding regions [32]; differences in data partitioning [33]; nucleotide substitution saturation [34], and gap percentages [35, 36]; and other causes. Therefore, we generated sequence matrices and performed phylogenetic analyses to evaluate the potential effects of these factors, as described below.

We generated three sequence matrices, PCG, NPCG (non-protein coding genes), and CP (complete plastomes with one inverted repeat region removed to avoid redundancy), to investigate potentially conflicting phylogenetic signals between coding and non-coding regions of the plastomes [32]. Initially, we concatenated loci to construct each matrix using the python script AMAS v1.0 [88], and we then manually edited the matrices with BioEdit v7.2.5 [89] when necessary.

Partitioning has been shown to improve phylogenetic inference in some studies [33] and has had no impact in others [32, 90]. We therefore implemented both partitioned and unpartitioned strategies on the three sequence matrices (CP, PCG, and NPCG) to determine the effects of partitioning. After generating the partition files in AMAS, we manually modified them into RAxML style, treating each locus as an independent data block. We used “-spp” in IQ-TREE v1.6.12 to determine the partition scheme [27, 91].

To evaluate the potential effect of nucleotide substitution saturation, we identified loci with high saturation levels using TreSpEx v1.1 [28]. The rationale of TreSpEx is that the larger the slope or R^2^ in a linear regression analysis of PD and uncorrected genetic distance (*p*), the less saturated the locus. Therefore, we first calculated PD and *p* based on the gene trees before performing linear regression in TreSpEx. We then plotted the distribution of slope and R^2^ values in R v3.6.2 [92]. We considered loci located on the shoulders of the slope or R^2^ plots as possibly saturated. Thus, we generated two new sequence matrices, CP-slope and CP-R^2^, with the potentially saturated loci removed.

As Duvall et al. [35] indicated, gaps in alignments can affect tree topology. Therefore, we trimmed sites above certain thresholds of gap percentage from the CP matrix using ClipKIT [93]. The trimming mode was set to “gappy”, and the threshold was set to 0.90 (removing sites with > 90% gap percentage), 0.70, 0.50, 0.25, 0.15, 0.14, 0.12, 0.10, 0.09, 0.07, 0.05, 0.04, 0.02, or 0 (including no gaps). The 14 gap-stripped matrices were used for ML analyses.

Combined, all of our strategies yielded 22 data matrices for analysis (Table 1). These included six matrices containing different plastid regions, with or without partitioning (CP, PCG, NPCG, CP-p, PCG-p, and NPCG-p). Among the 22 matrices, two (CP-slope and CP-R^2^) were generated by removing probably saturated loci. The remaining 14 matrices were generated by trimming gapped sites of CP that were above specific thresholds of gap percentage (“CP-gappy” in Table 1).

For all 22 matrices, we determined alignment length, number of gaps, parsimony-informative sites, and AT content using AMAS. We plotted the variation of gap abundance across loci in R. For each matrix or partition, we determined the best-fitting models of nucleotide substitution in ModelFinder [94] under the Bayesian Information Criterion (BIC) implemented in IQ-TREE. We used IQ-TREE to perform ML analyses with 1000 replicates each of UFBS [95] and the SH-aLRT [96] to determine node support. In all analyses, we applied the ‘-bnni’ parameter to reduce the risk of overestimating branch support due to any possible severe model violations [27].

### Divergence time estimation

For divergence time estimation, we downloaded an additional 17 plastomes representing other major clades of Lauraceae (Table S7). The large volume of plastid data could make divergence-time estimation intractable, and among loci, topology and substitution rate heterogeneity may lead to model violations. Thus, we applied the gene-shopping method to filter protein-coding genes using SortaDate [97] based on three principles: clock-likeness, reasonable tree length, and least topological conflict with the species tree (the unpartitioned CP ML tree; Fig. 1).

We performed molecular dating analysis in BEAST v2.6.3 [30]. For the analysis, we concatenated the selected loci, used BEAUti [30] to import them, set the substitution model to GTR, implemented a relaxed log-normal molecular clock, and applied a Yule model for the tree branching process with birth rate set to a gamma distribution with an alpha and beta of 0.001 and 1000, respectively. We ran the analysis for 200,000,000 Markov chain Monte Carlo (MCMC) generations, sampling every 20,000 generations. Following the analysis, we imported the log file into Tracer v1.7.1 [98] to verify stationarity based on all estimated parameters with ESS ≥ 200. After removing the first 20% of trees as burn-in, we generated a maximum clade credibility (MCC) tree in TreeAnnotator v2.6.3 [30] and visualized it using FigTree v1.4.3 [99].

We used a secondary calibration point and three macrofossils for divergence time estimation. For the secondary calibration, we set a normal prior with an offset of 98 Ma, a mean of 0, and a sigma of 1.0 for the crown age of Lauraceae following Nie et al. [100] and Li et al. [101]. One of the macrofossils, *Neusenia tetrasporangiata* Eklund, is a well-preserved flower bud from the Santonian/Campanian (ca. 83 Ma; late Cretaceous) in the Neuse River locality in North Carolina, USA, and it can be assigned to *Neocinnamomum* H. Liu [102, 103]. Therefore, we used this fossil to set a log-normal prior with an offset of 83 Ma, a mean of 1.0, and a standard deviation of 0.6 for the crown age of *Neocinnamomum*-*Caryodaphnopsis*-core Lauraceae following the strategy of Li et al. [104].

Two leaf macrofossils are also highly relevant to divergence time dating in tribe Perseeae: *Alseodaphne changchangensis* Jin & Li, which was found in the Upper Member, or rock bed assemblage, of the Eocene Changchang Formation of the Changchang Basin within Hainan Province, China [105], and *Machilus maomingensis* Jin & Tang, which was found in the Upper Member of the middle/late Eocene Youganwo Formation outcropping in the Maoming Basin of Guangdong Province, China [106]. Determining the genera of fossils from Lauraceae and extant groups is difficult based only on isolated leaves [107], unless the species is clearly recognizable [44]. Thus, based on the CP ML tree (Fig. 1), there were several options for the placement of *A. changchangensis* and *M. maomingensis* for age calibration. We considered three nodes (nodes 2, 3 and 4 in Fig. 2b and S11) as candidates for calibration points. In scenarios 1, 2, and 3, we placed *A. changchangensis* at nodes 2, 3, and 4, respectively (Table S3). In scenarios 4 and 5, we fixed *M. maomingensis* at nodes 2 and 3, respectively (Table S3).

Fossil fruit cupules attributable to the Perseeae + Laureae clade are abundant in the London Clay Flora, which represents the early Eocene beginning 56 Ma [62]. Thus, the stratigraphic age of London Clay Flora has been considered to be the minimum stem age of tribe Perseeae, which was estimated to be 55.3 Ma (95% HPD = 41.4–69.9 Ma) by Li et al. [21] using molecular dating. Therefore, we ran the dating scenarios with the different calibration points for *A. changchangensis* and *M. maomingensis* and determined their placement based on the result showing a stem age for the tribe closest to 55.3 Ma. We then used the inferred best calibrations for both fossils in a final dating scenario, Scenario 6 (Table S3).

In scenarios 1–6, we fixed the position of *N. tetrasporangiata* and the secondary calibration point at nodes 5 and 6, respectively (Table S3; Fig. S11). For *A. changchangensis*, we applied a log-normal prior distribution with an offset of 37 Ma, a mean of 1.0 and, a standard deviation of 0.85, and for *M. maomingensis*, we used a log-normal prior distribution with an offset of 33.7 Ma, a mean of 1, and a standard deviation of 0.85. In the molecular dating analyses, we found that the phylogenetic positions of several species of *Phoebe* had changed, although they were strongly supported in the ML tree (Fig. 1). This may have been caused by reduced numbers of parsimony informative sites in the three selected loci. To reduce their influence on tree topology and downstream analyses, we constrained the phylogenetic positions of these *Phoebe* species in scenarios 1–6.

### Diversification rate analysis

Although no method of diversification rate analysis is entirely robust to poor taxon sampling, BAMM v2.5 [31] has been shown to be less sensitive than RPANDA [108] and the DR statistic [109] with a moderate level of missing taxa [53]. We, therefore, employed BAMM to infer speciation rates and possible rate shifts across the dated MCC tree. Before applying BAMM, we removed outgroup taxa using APE v5.4-1 [110] and determined priors with BAMMtools v2.1.6 [111] in R. The prior for “ExpectedNumberofShifts” was set to 1 because the tip number of the MCC tree was smaller than 500 [111]. To account for missing extant taxa, we specified the global sampling probability as 0.135. We ran BAMM for 10,000,000 generations and sampled every 1000 generations. Following the analysis, we calculated the ESS of loglikelihood and number of shifts to evaluate convergence based on ESS ≥ 200 using the CODA R package [112]. Thereafter, we analyzed BAMM output files using BAMMtools with 25% of samples discarded as burn-in. From the remaining samples, we inferred the 95% credible set of macroevolutionary rate shift configurations according to BAMM posterior probability, and we used this to plot speciation rate, extinction rate, and net diversification rate through time in BAMMtools. Within the plots, we included relative global temperature change during the past 45 million years (Fig. 3a) according to Zachos et al. [113].

Although we have collected 365 accepted species names of tribe Perseeae (Table S1), the true diversity of this tribe may be larger, because of inadequate investigation and taxonomic difficulties. To test the sensitivity of BAMM analysis to inaccurate estimates of diversity in tribe Perseeae and incomplete sampling, we ran an additional analysis using BAMM with global sampling probability halved and other prior parameters unchanged, following Shi and Rabosky [52].

To evaluate the robustness of the diversification pattern inferred by BAMM, we also performed an analysis using CoMET within the TESS R package [114]. Before running CoMET, we specified the prior distribution of model parameters following Höhna et al. [115], and we used the same sampling probability and input tree as in the BAMM analysis. We ran the CoMET analysis for 4,000,000 iterations, with 400,000 iterations discarded as burn-in. We summarized and visualized the results using the tess.process.output and tess.plot.output functions, respectively. To ensure reliable estimates, we performed single-chain diagnostics to evaluate the ESS and Geweke diagnostic for diversification rates, shift times, and mass extinction events.

### Ancestral habitat

We determined the geographic distribution of all sampled species of tribe Perseeae based on the National Specimen Information Infrastructure (NSII) [116] and the Flora of China [18] and used the distributions to assess their vegetation types [15, 117]. Based on the results, we identified three vegetation types and coded species according to their presence in each: (1) tropical forests, (2) East Asian subtropical EBLFs, and (3) evergreen forests of the southeastern United States of America (U.S.A.). Tropical forests in Asia and the Americas were included in the same category. We coded species found in both tropical forests and subtropical EBLFs as an additional unique state.

As incomplete sampling of outgroups can bias ASR, we excluded all outgroups before analysis [13, 118]. We reconstructed the ancestral habitat of tribe Perseeae using SIMMAP [9], a Bayesian approach implemented in the R package phytools [119]. For the analysis, we selected the best-fitting model from among “equal-rates” (ER), “symmetric” (SYM), and “all-rates-different” (ARD) according to the AIC using the function fitDiscrete in the R package geiger [120]. Within SIMMAP, we set the number of simulations to 200, and we visualized the analysis results in the ggtree R package [121].

## Supplementary Information

**Additional file 1: Fig. S1.** Complete plastid genome map of tribe Perseeae. Different genes are color coded.

**Additional file 2: Fig. S2.** ML tree inferred from IQ-TREE based on unpartitioned protein coding genes (PCG). The support values of Shimodaira-Hasegawa-like approximate likelihood ratio test (SH-aLRT; on the left) and ultrafast bootstrap (UFBS; on the right) are shown on the branches, respectively.

**Additional file 3: Fig. S3.** ML tree inferred from IQ-TREE based on unpartitioned non-protein coding genes (NPCG). The support values of SH-aLRT (on the left) and UFBS (on the right) are shown on the branches.

**Additional file 4: Fig. S4.** ML tree inferred from IQ-TREE based on partitioned genes of complete plastomes (CP). The support values of SH-aLRT (on the left) and UFBS (on the right) are shown on the branches.

**Additional file 5: Fig. S5.** ML tree inferred from IQ-TREE based on partitioned PCG. The support values of SH-aLRT (on the left) and UFBS (on the right) are shown on the branches.

**Additional file 6: Fig. S6.** ML tree inferred from IQ-TREE based on partitioned NPCG. The support values of SH-aLRT (on the left) and UFBS (on the right) are shown on the branches.

**Additional file 7: Fig. S7.** Saturation indices for 197 loci shown as density plots. (a) Slopes of the linear regression between patristic and uncorrected pairwise distances. (b) R^2^ of the linear regression between patristic and uncorrected pairwise distances. The vertical and dashed lines indicate starting shoulder value. Loci that may be saturated are colored in red; the numbers of saturated loci are indicated aside.

**Additional file 8: Fig. S8.** ML tree inferred from IQ-TREE based on unpartitioned CP-slope. CP-slope refers to CP after excluding saturated loci based on slopes of the linear regression. The support values of SH-aLRT (on the left) and UFBS (on the right) are shown on the branches, respectively.

**Additional file 9: Fig. S9.** ML tree inferred from IQ-TREE based on unpartitioned CP-R^2^. CP-R^2^ refers to CP after excluding saturated loci based on R^2^ of the linear regression. The support values of SH-aLRT (on the left) and UFBS (on the right) are shown on the branches.

**Additional file 10: Fig. S10.** Number of gaps in each locus. The locus with the highest number of gaps (*ccsA-trnL*^*UAG*^) is marked.

**Additional file 11: Fig. S11.** Divergence times inferred from BEAST2. The red pentagrams refer to the fossil and secondary calibration points. The white numbers in black circles correspond to those in Fig. 2 and mentioned in the main text. The numbers and blue bars at nodes are divergence times before present and corresponding time intervals in the 95% highest posterior density (HPD).

**Additional file 12: Fig. S12.** Credible shift set inferred from BAMM analysis. The values of f indicate the probabilities of speciation rate shifts in the maximum sampled *posterior* configuration.

**Additional file 13: Fig. S13.** Speciation rate and location of rate shift when global sampling probability was 0.0675.

**Additional file 14: Fig. S14.** Diversification rate inferred from CoMET in TESS. The shaded areas in (a) and (c) indicate 95% confidence intervals of speciation and extinction rates. 2lnBF (the heights of bars) higher than 6 indicate significant speciation rate shift, extinction rate shift, or mass extinction in (b), (d), and (e).

**Additional file 15: Fig. S15.** The single-chain MCMC diagnostics for a CoMET analysis. The blue bars and dots indicate passed tests, while red bars and dots refer to failed convergence.

**Additional file 16: Table S1.** The species names of tribe Perseeae.

**Additional file 17: Table S2.** Characteristics of the 242 loci of tribe Perseeae and outgroups.

**Additional file 18: Table S3.** The stem age of tribe Perseeae under different scenarios. The node numbers in the table correspond to node numbers in Figs. 2 and S12.

**Additional file 19: Table S4.** The vegetation types of 52 sampled species in tribe Perseeae. Tropical forests, subtropical EBLFs, southeastern evergreen forests in North America are coded as 0, 1, and 2, respectively. Species distributed in both tropical forests and subtropical EBLFs is coded as 3.

**Additional file 20: Table S5.** Model selection results inferred from geiger R package. AIC, Akaike Information Criterion.

**Additional file 21: Table S6.** The newly sequenced species of tribe Perseeae and outgroups.

**Additional file 22: Table S7.** The downloaded plastomes of Lauraceae.

## Data Availability

Plastomes generated in this study are released in the Science Data Bank (https://www.scidb.cn/) [122] and NCBI (https://www.ncbi.nlm.nih.gov/nuccore/) [84], with GenBank accession numbers shown in Table S6. Sequencing data are deposited in Sequence Read Archive of NCBI (https://www.ncbi.nlm.nih.gov/bioproject/PRJNA756055), the SRA accession numbers are SRR15566192-SRR15566234 (Table S6). All the sequence matrices in this study, partition files, and generated tree files are deposited at Dryad repository [29]. Public access to the databases mentioned above are open and no administrative permissions are needed for accessing and using the data. Material samples are available from authors. Voucher specimens were deposited in the herbarium of the South China Botanical Garden (IBSC) (Table S6).

## Abbreviations

AIC: Akaike Information Criterion
ASR: Ancestral state reconstruction
BAMM: Bayesian analysis of macro-evolutionary mixtures
BIC: Bayesian Information Criterion
CP: Complete plastomes
CoMET: CPP on Mass-Extinction Times
CTAB: cetyl trimethyl ammonium bromide
EAM: East Asian monsoon
EBLFs: Evergreen broad-leaved forests
ESS: Effective sample size
HPD: Highest posterior density
IBSC: The herbarium of South China Botanical Garden, Chinese Academy of Sciences
IGS: Intergenic spacers
ILS: Incomplete lineage sorting
IR: Inverted repeat region
LSC: Large single-copy region
MCC: Maximum clade credibility
ML: Maximum likelihood
MRCA: Most recent common ancestor
NCBI: National Center for Biotechnology Information
NPCG: Non-protein coding genes
nrDNA: Nuclear ribosomal DNA
NSII: National Specimen Information Infrastructure
O-M: Oligocene-Miocene
OGDRAW: OrganellarGenomeDRAW
PCG: Protein coding genes
PD: Patristic distance
rRNA: Ribosomal RNA
SH-aLRT: Shimodaira-Hasegawa-like approximate likelihood ratio test
SSC: Small single-copy region
tRNA: Transfer RNA
UFBS: Ultrafast bootstrap
