# Supplementary figures and images for: Plastid phylogenomics of tribe Perseeae (Lauraceae) yields insights into the evolution of East Asian subtropical evergreen broad-leaved forests
Source: BMC Plant Biol. 2022 Jan 13;22:32. doi: 10.1186/s12870-021-03413-8 (PMC8756638; doi:10.1186/s12870-021-03413-8)

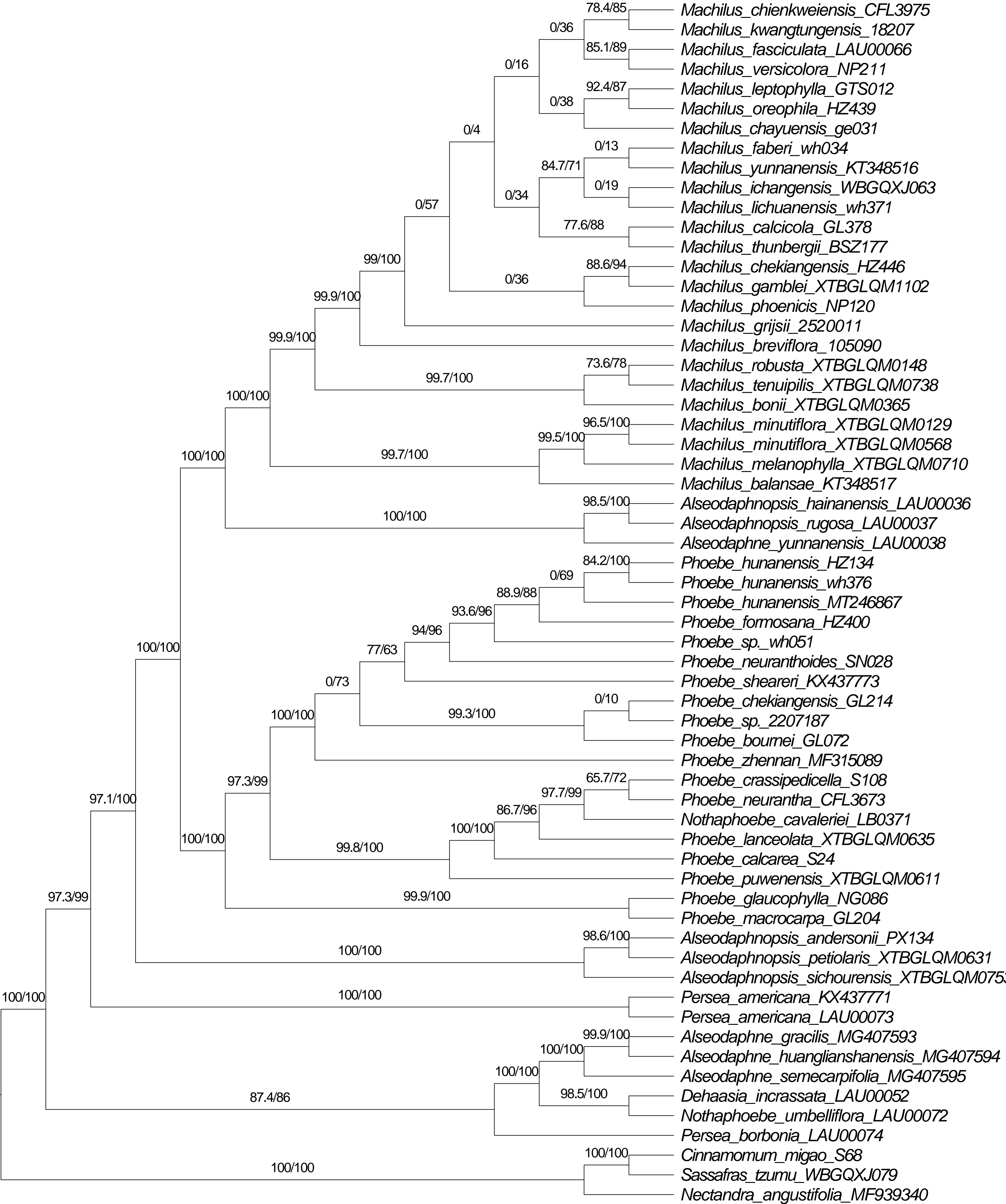

Supplement: Supplementary file 2 — Additional file 2: Fig. S2. ML tree inferred from IQ-TREE based on unpartitioned protein coding genes (PCG). The support values of Shimodaira-Hasegawa-like approximate likelihood ratio test (SH-aLRT; on the left) and ultrafast bootstrap (UFBS; on the right) are shown on the branches, respectively. [file 12870_2021_3413_MOESM2_ESM.pdf]

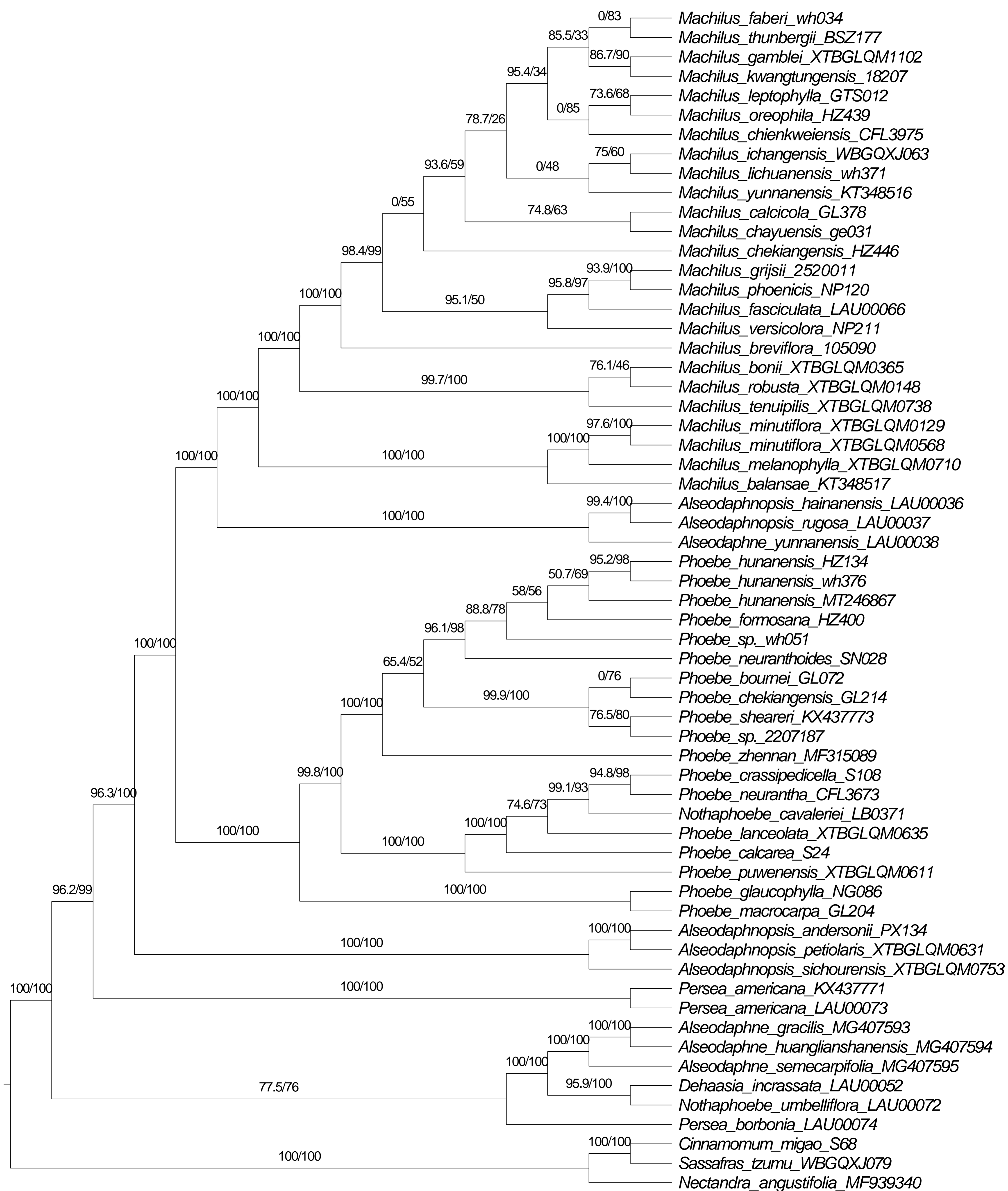

Supplement: Supplementary file 3 — Additional file 3: Fig. S3. ML tree inferred from IQ-TREE based on unpartitioned non-protein coding genes (NPCG). The support values of SH-aLRT (on the left) and UFBS (on the right) are shown on the branches. [file 12870_2021_3413_MOESM3_ESM.pdf]

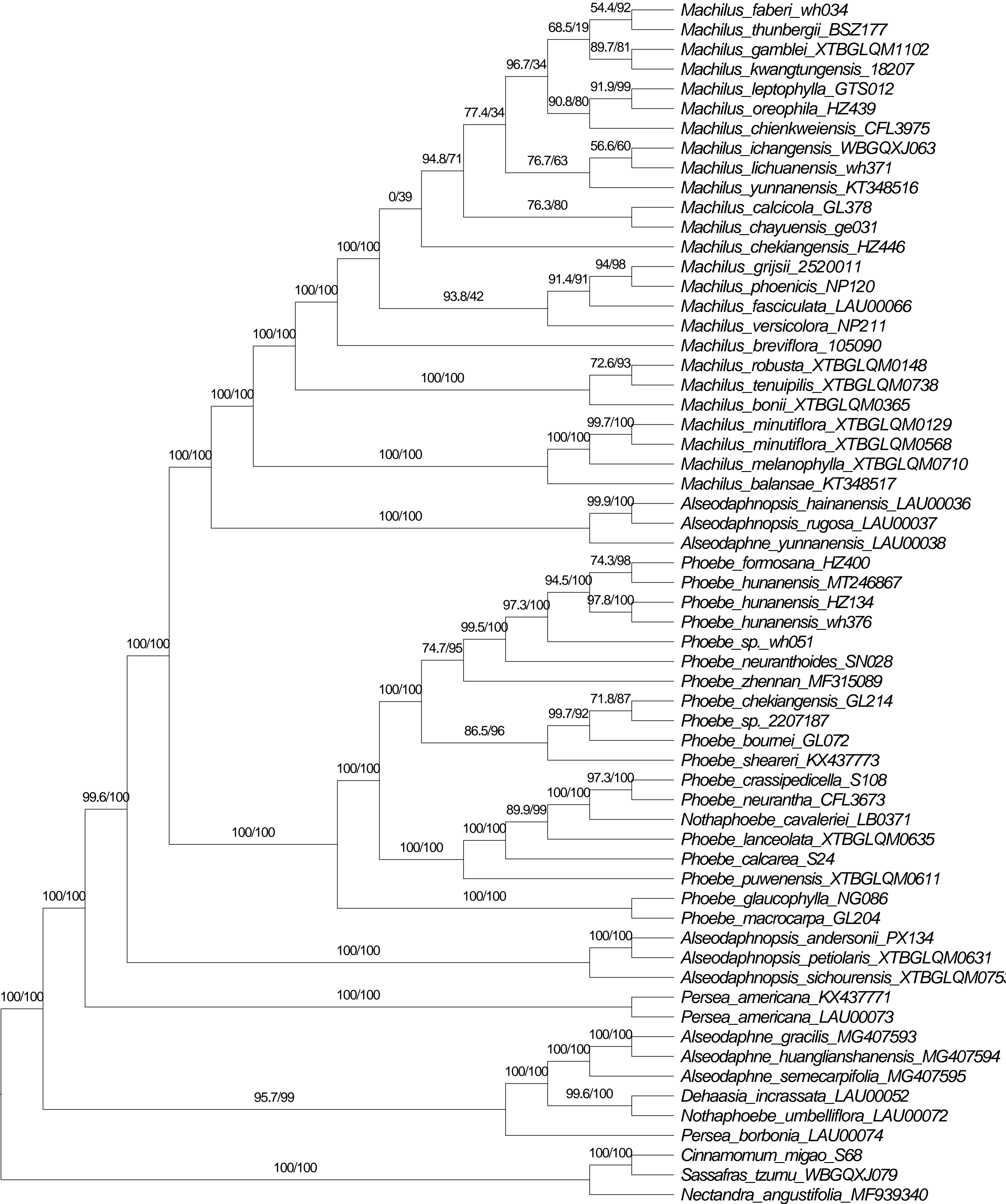

Supplement: Supplementary file 4 — Additional file 4: Fig. S4. ML tree inferred from IQ-TREE based on partitioned genes of complete plastomes (CP). The support values of SH-aLRT (on the left) and UFBS (on the right) are shown on the branches. [file 12870_2021_3413_MOESM4_ESM.pdf]

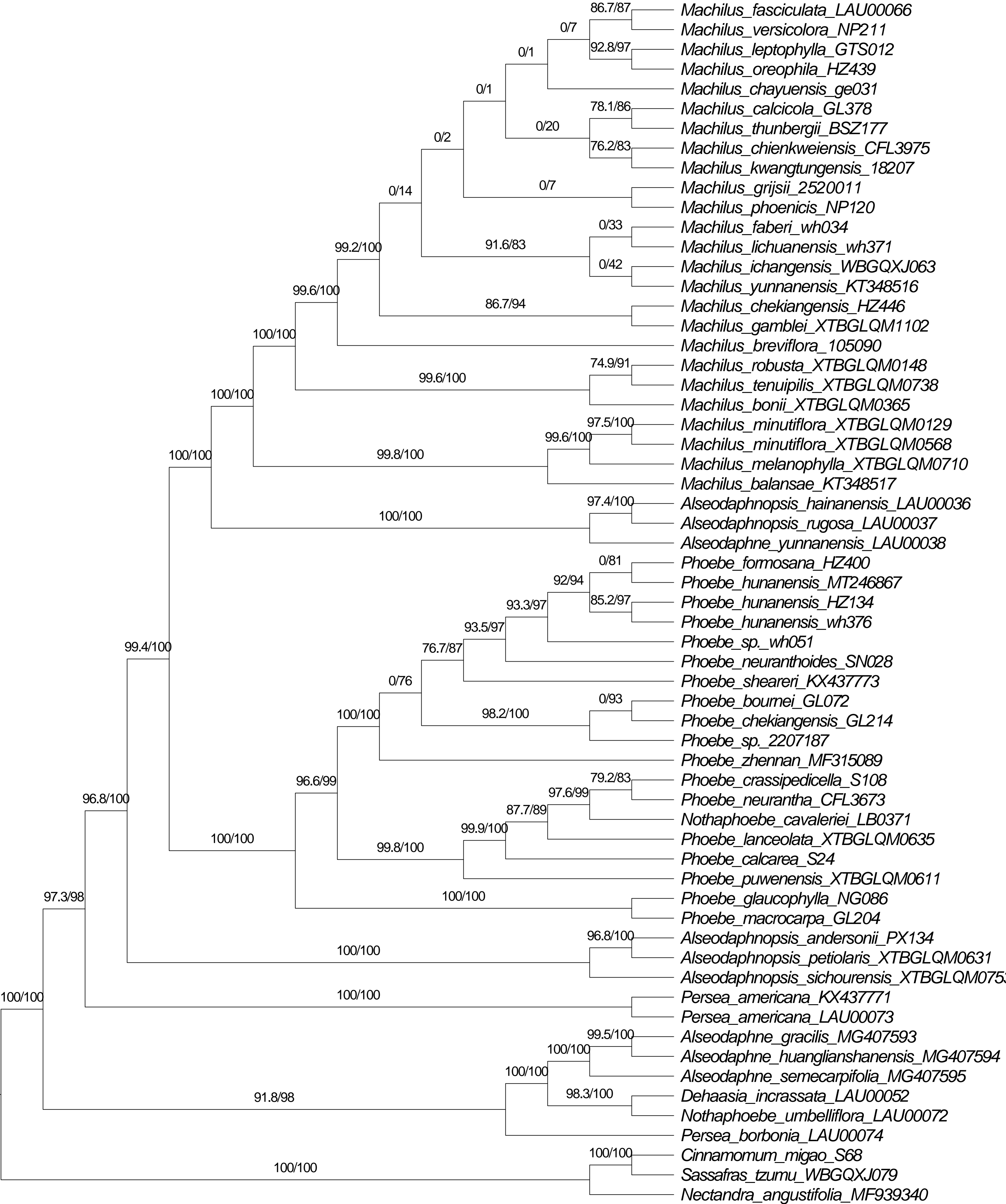

Supplement: Supplementary file 5 — Additional file 5: Fig. S5. ML tree inferred from IQ-TREE based on partitioned PCG. The support values of SH-aLRT (on the left) and UFBS (on the right) are shown on the branches. [file 12870_2021_3413_MOESM5_ESM.pdf]

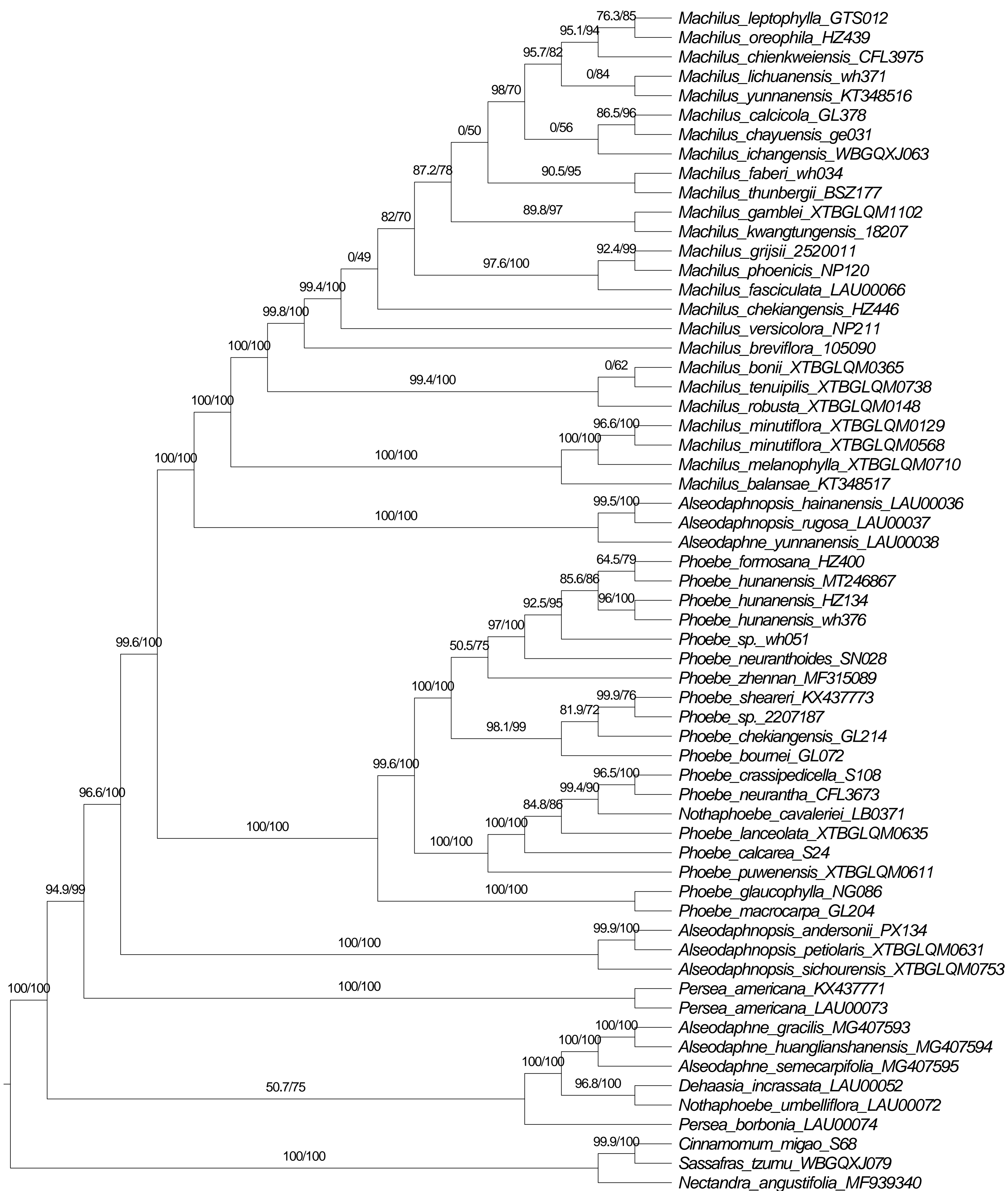

Supplement: Supplementary file 6 — Additional file 6: Fig. S6. ML tree inferred from IQ-TREE based on partitioned NPCG. The support values of SH-aLRT (on the left) and UFBS (on the right) are shown on the branches. [file 12870_2021_3413_MOESM6_ESM.pdf]

a)

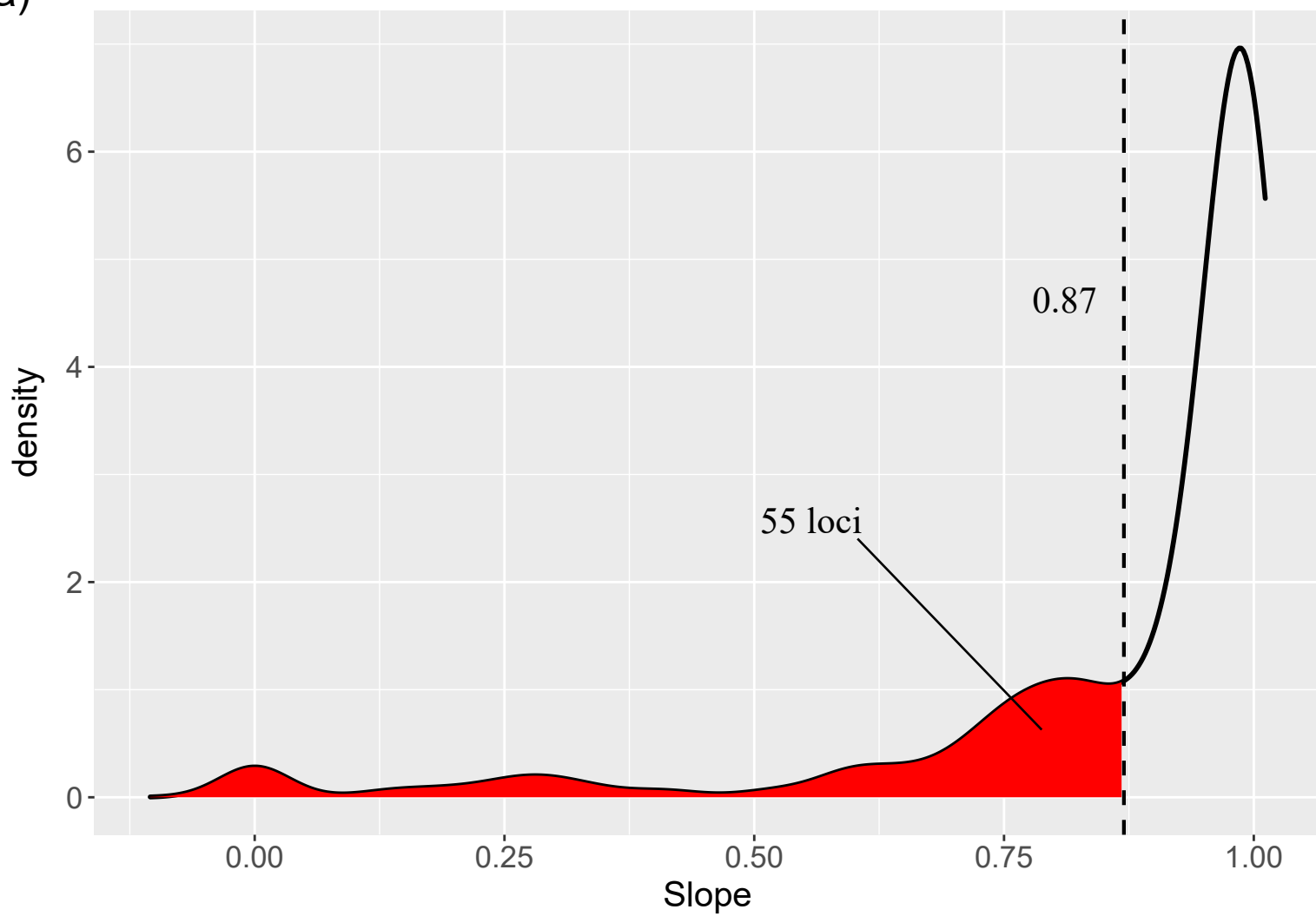

b)

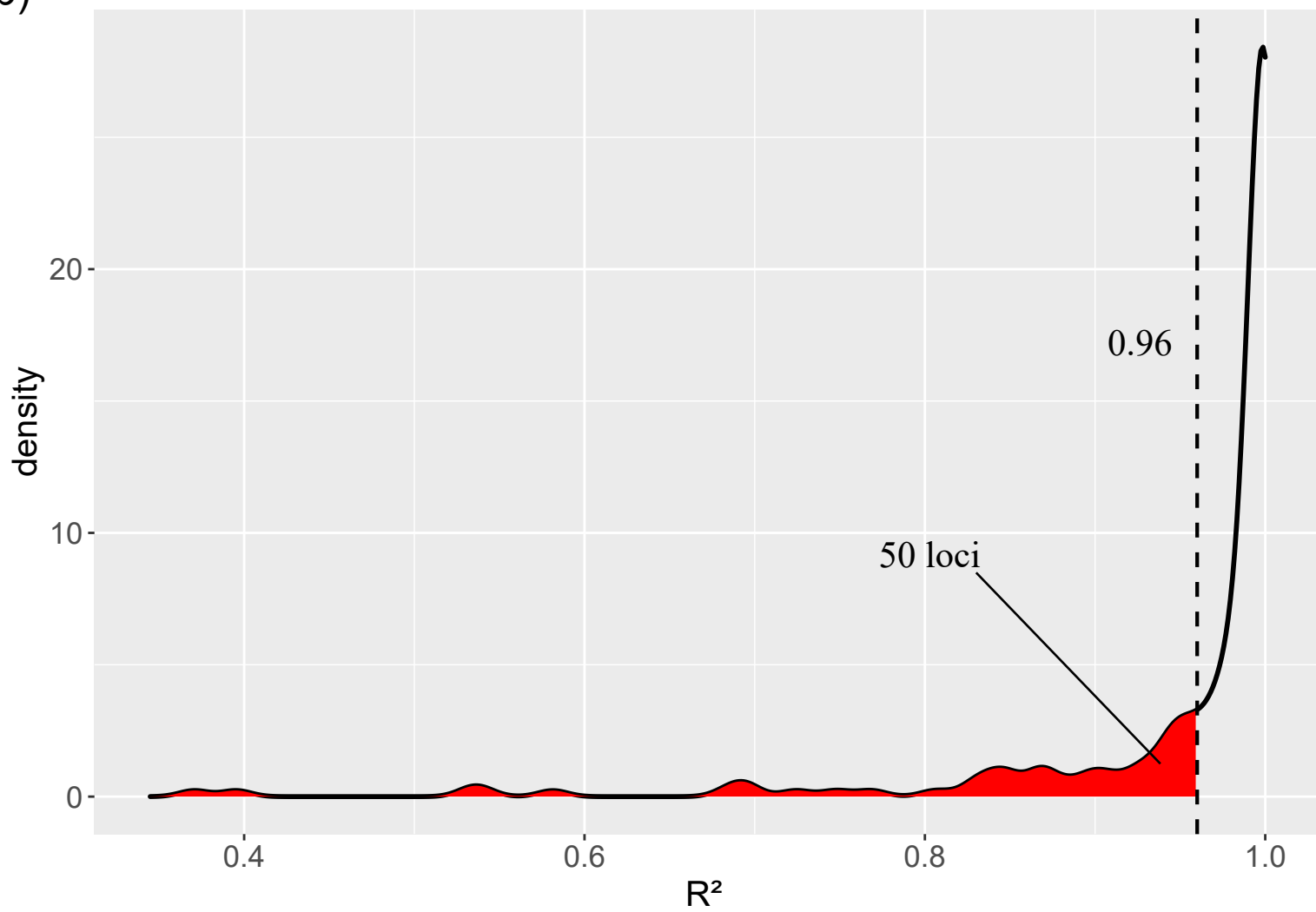

Supplement: Supplementary file 7 — Additional file 7: Fig. S7. Saturation indices for 197 loci shown as density plots. (a) Slopes of the linear regression between patristic and uncorrected pairwise distances. (b) R2 of the linear regression between patristic and uncorrected pairwise distances. The vertical and dashed lines indicate starting shoulder value. Loci that may be saturated are colored in red; the numbers of saturated loci are indicated aside. [file 12870_2021_3413_MOESM7_ESM.pdf]

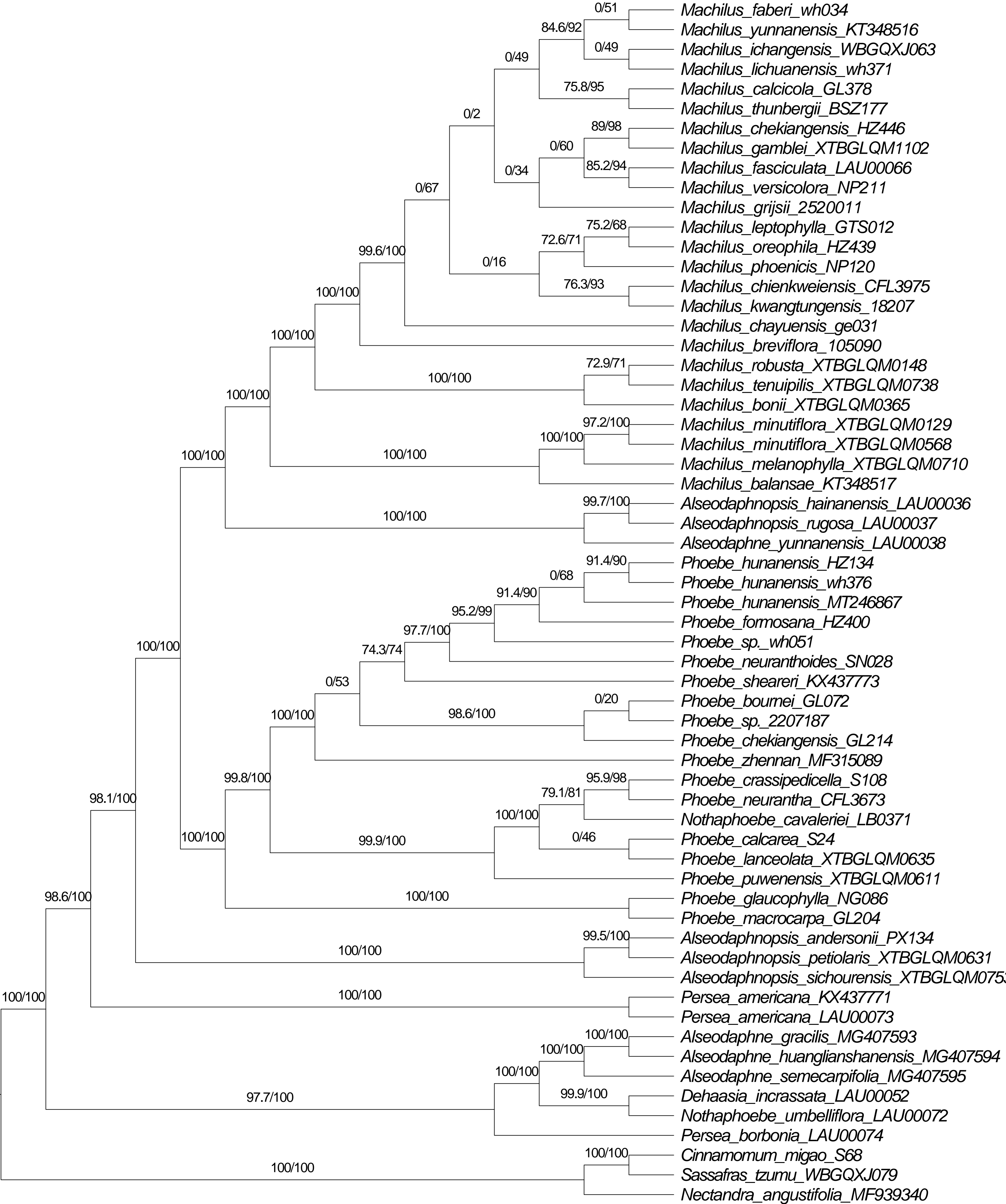

Supplement: Supplementary file 8 — Additional file 8: Fig. S8. ML tree inferred from IQ-TREE based on unpartitioned CP-slope. CP-slope refers to CP after excluding saturated loci based on slopes of the linear regression. The support values of SH-aLRT (on the left) and UFBS (on the right) are shown on the branches, respectively. [file 12870_2021_3413_MOESM8_ESM.pdf]

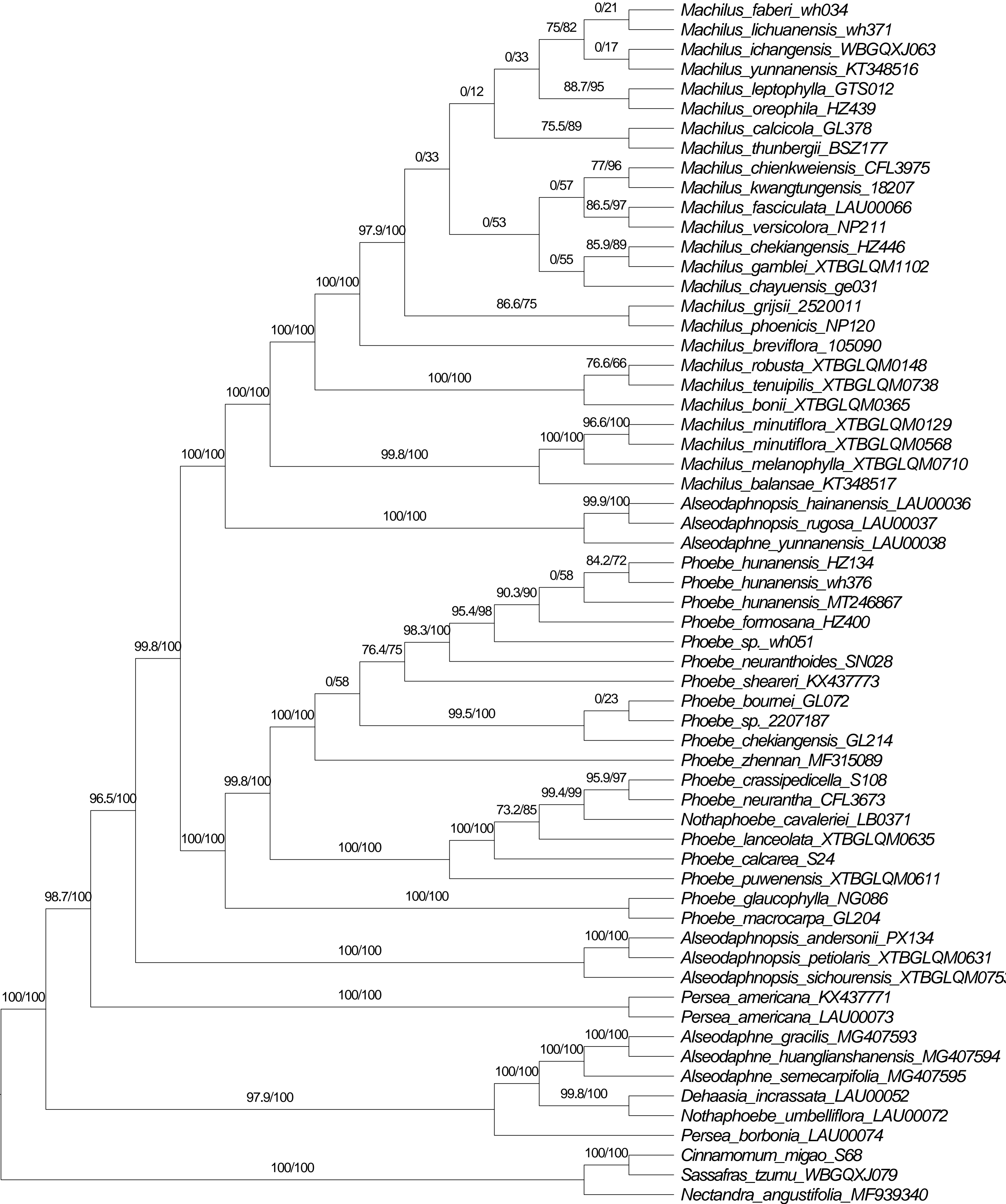

Supplement: Supplementary file 9 — Additional file 9: Fig. S9. ML tree inferred from IQ-TREE based on unpartitioned CP-R2. CP-R2 refers to CP after excluding saturated loci based on R2 of the linear regression. The support values of SH-aLRT (on the left) and UFBS (on the right) are shown on the branches. [file 12870_2021_3413_MOESM9_ESM.pdf]

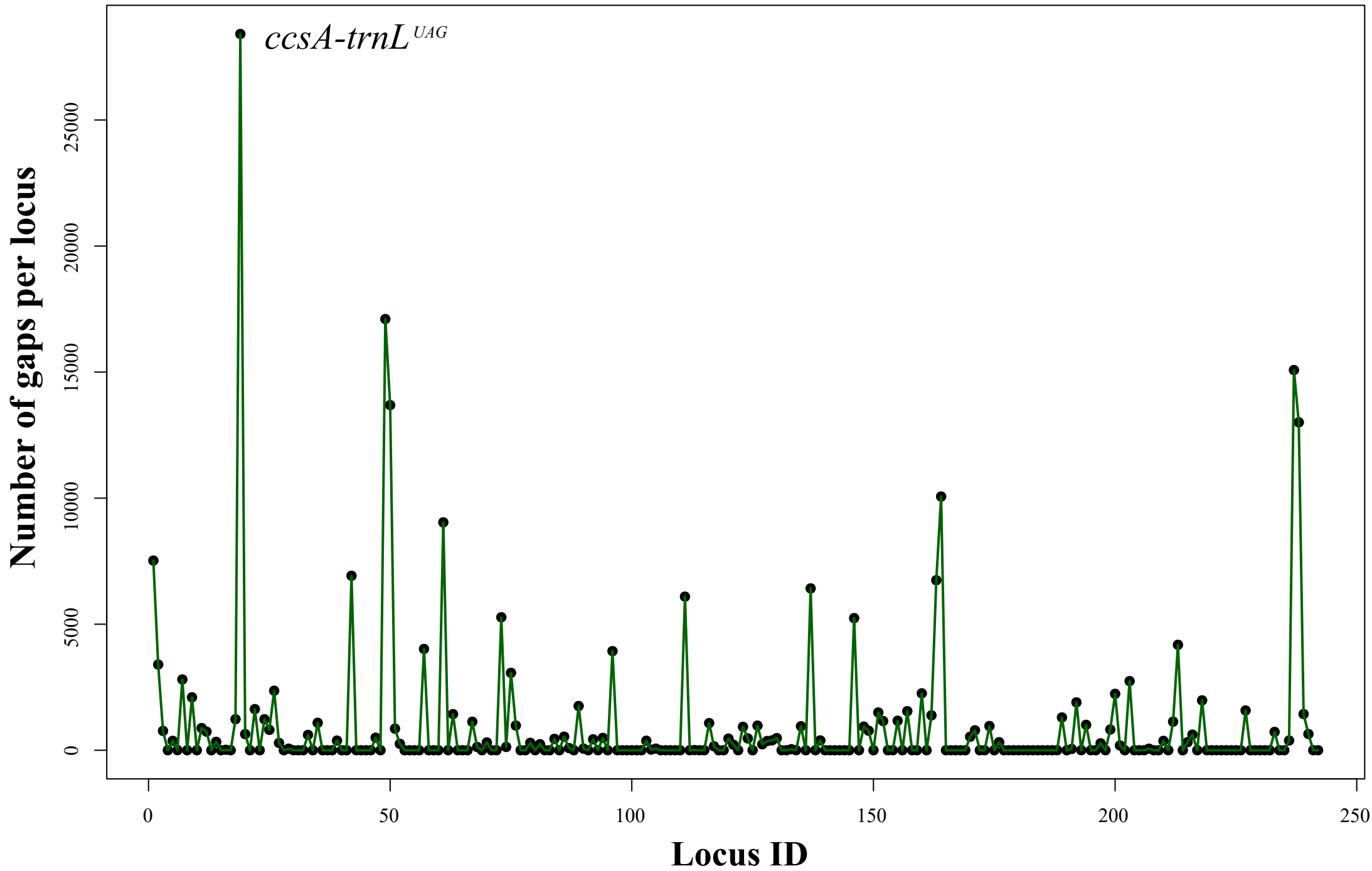

Supplement: Supplementary file 10 — Additional file 10: Fig. S10. Number of gaps in each locus. The locus with the highest number of gaps (ccsA-trnLUAG) is marked. [file 12870_2021_3413_MOESM10_ESM.pdf]

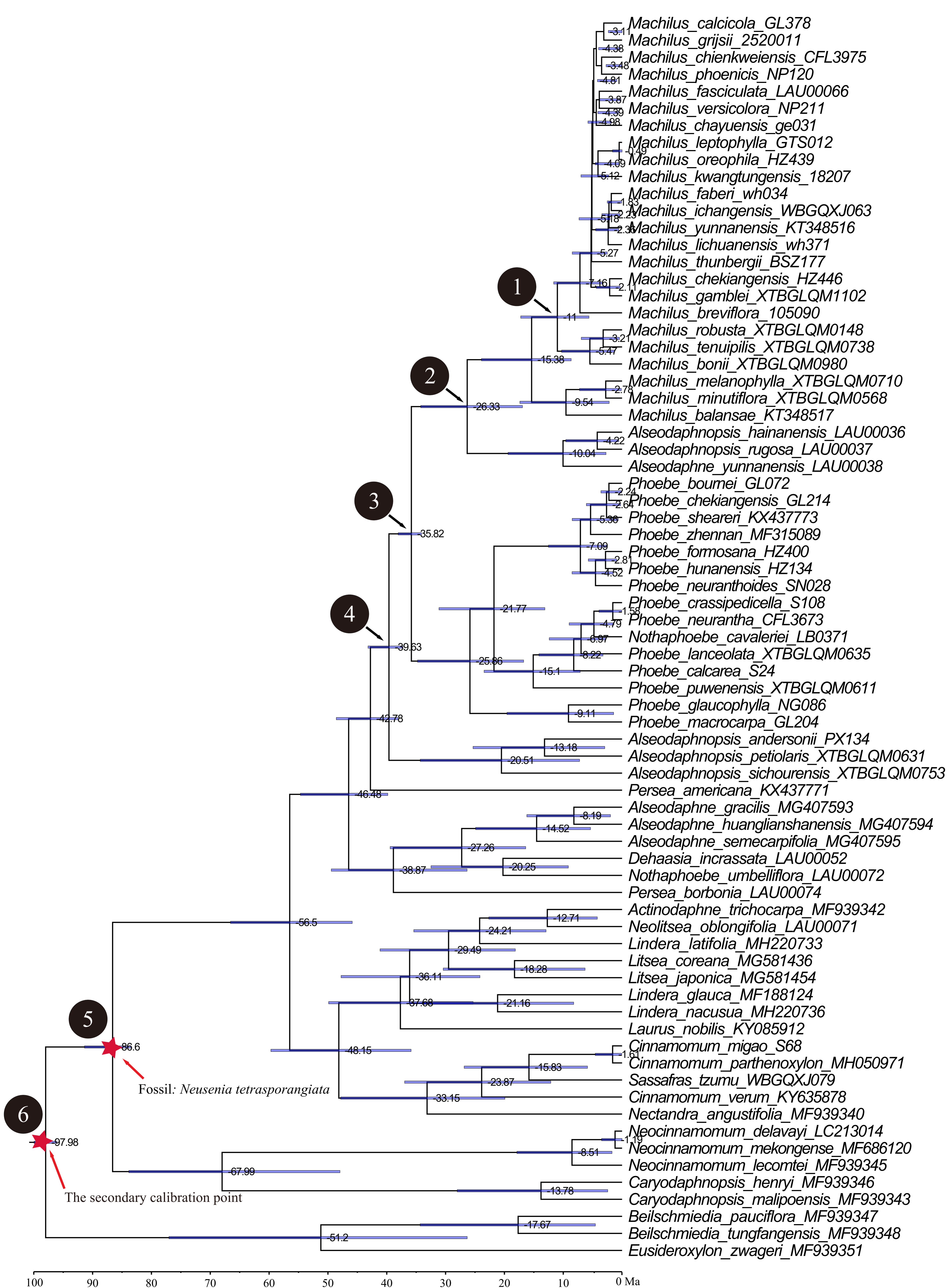

Supplement: Supplementary file 11 — Additional file 11: Fig. S11. Divergence times inferred from BEAST2. The red pentagrams refer to the fossil and secondary calibration points. The white numbers in black circles correspond to those in Fig. 2 and mentioned in the main text. The numbers and blue bars at nodes are divergence times before present and corresponding time intervals in the 95% highest posterior density (HPD). [file 12870_2021_3413_MOESM11_ESM.pdf]

$f = 0.29$

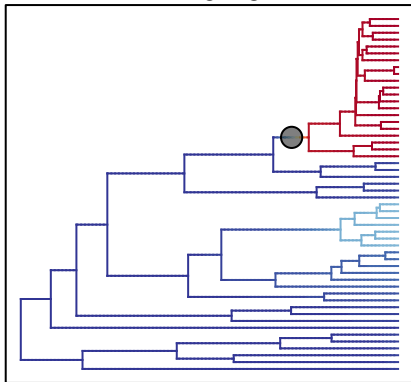

$f = 0.19$

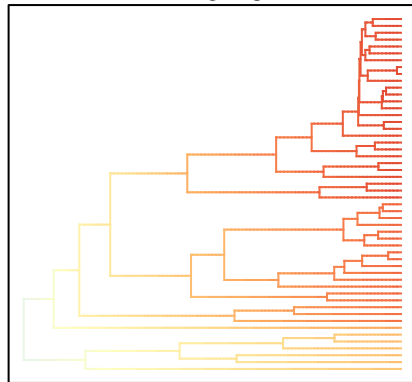

$f = 0.18$

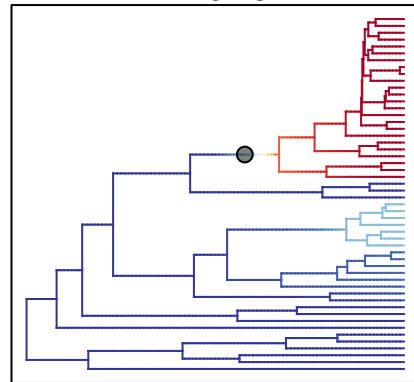

$f = 0.14$

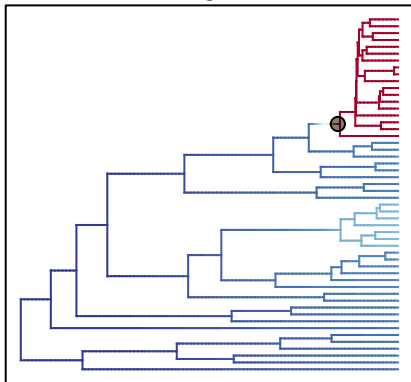

$f = 0.11$

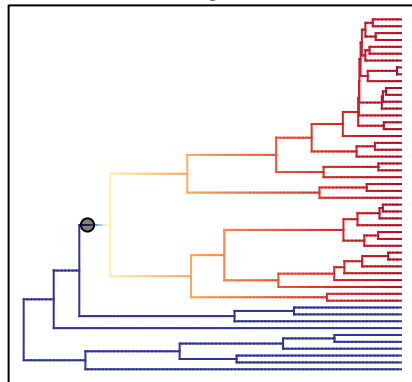

$f = 0.074$

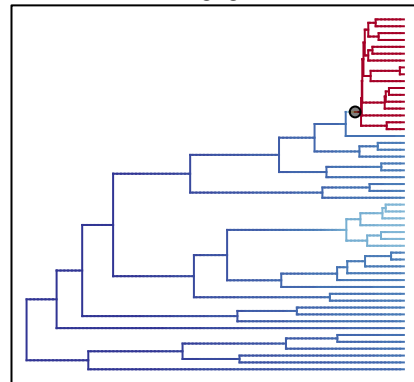

Supplement: Supplementary file 12 — Additional file 12: Fig. S12. Credible shift set inferred from BAMM analysis. The values of f indicate the probabilities of speciation rate shifts in the maximum sampled posterior configuration. [file 12870_2021_3413_MOESM12_ESM.pdf]

Sampling probability = 0.0675

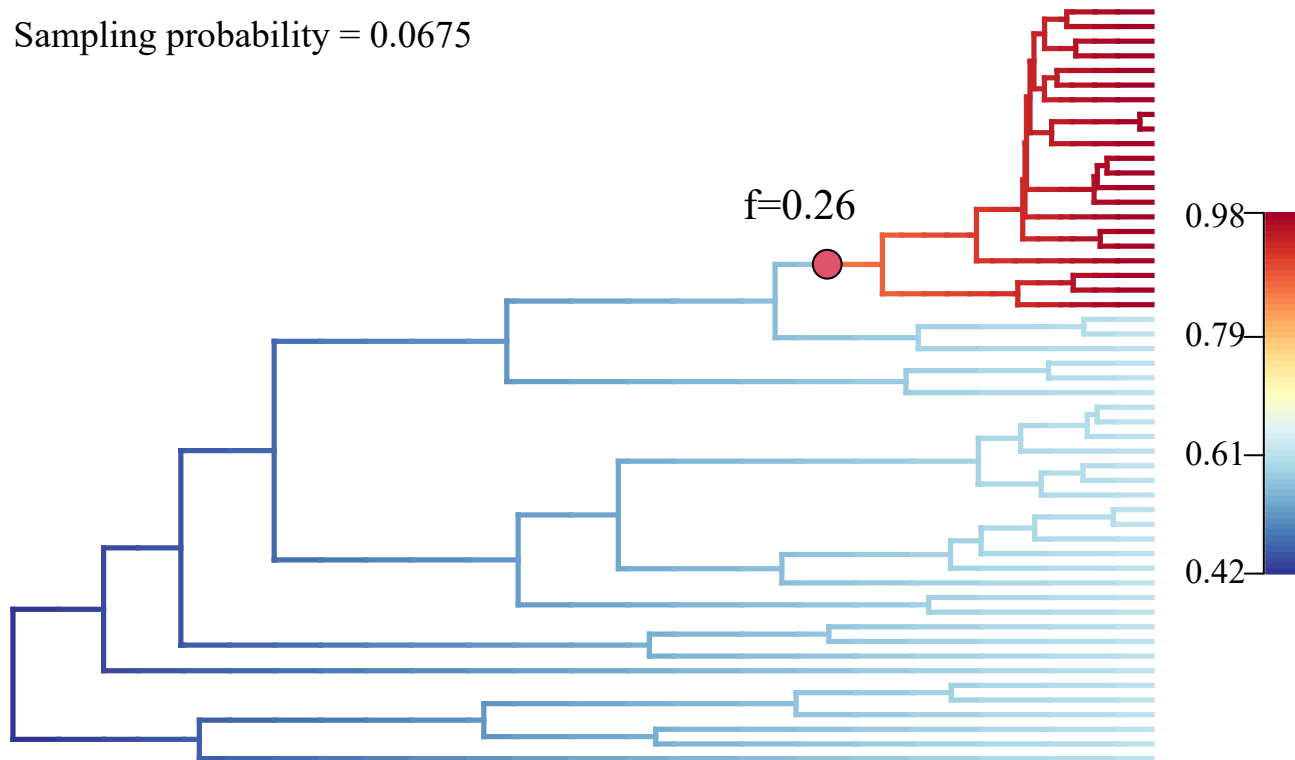

Supplement: Supplementary file 13 — Additional file 13: Fig. S13. Speciation rate and location of rate shift when global sampling probability was 0.0675. [file 12870_2021_3413_MOESM13_ESM.pdf]

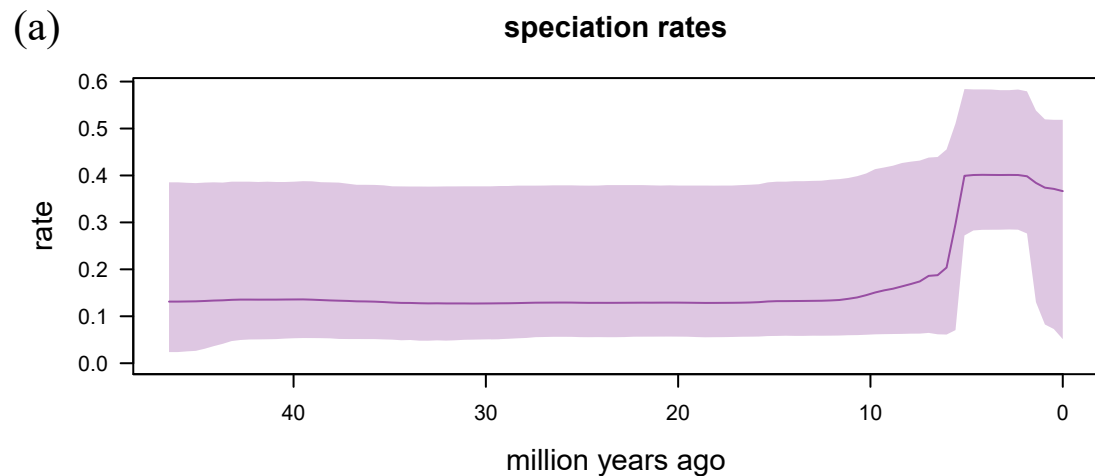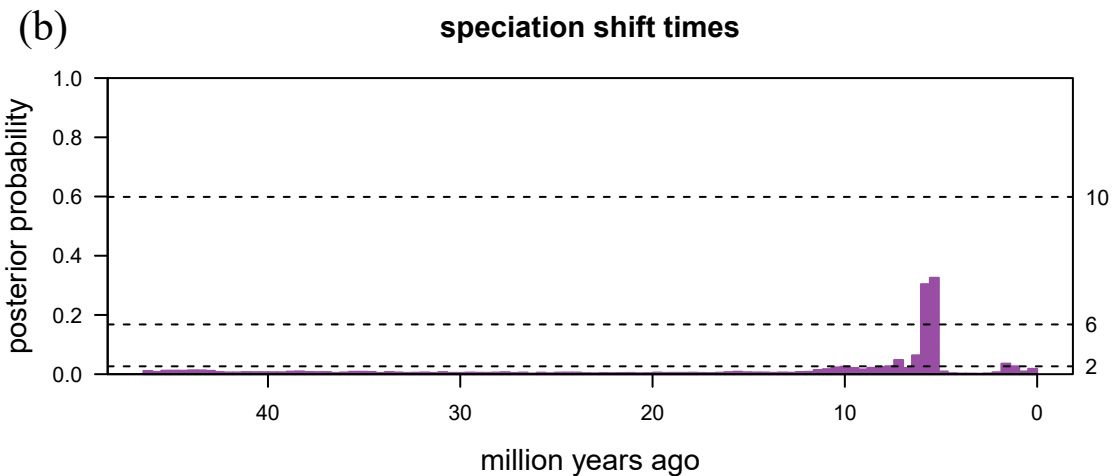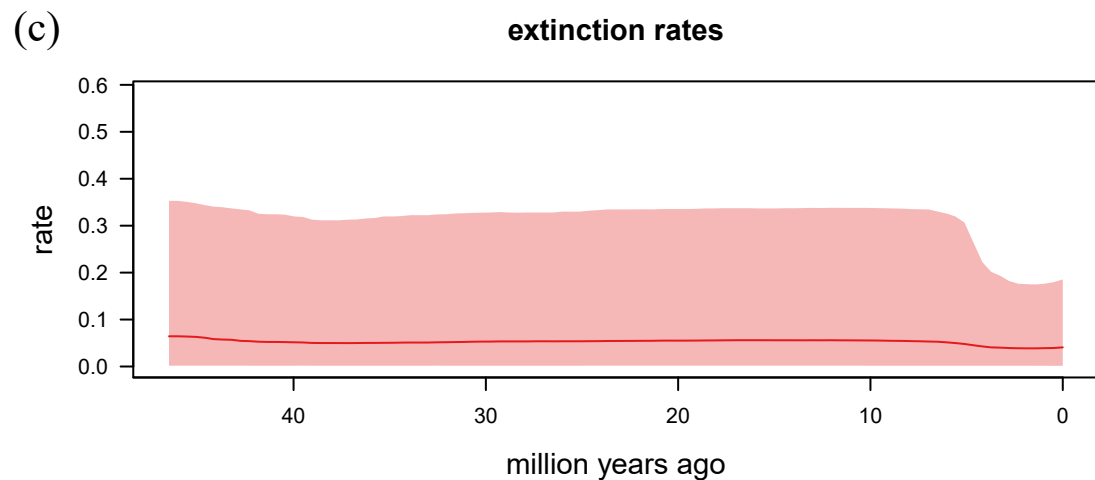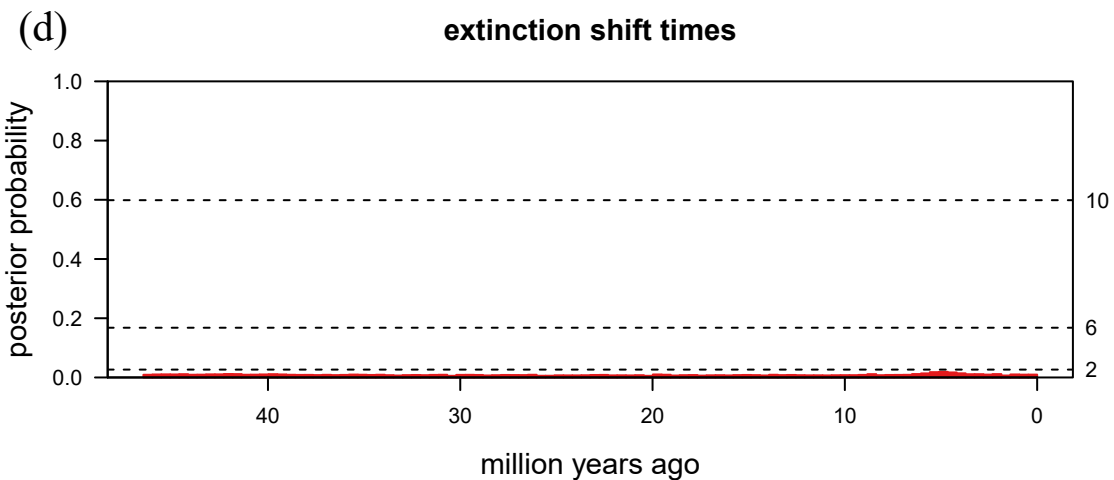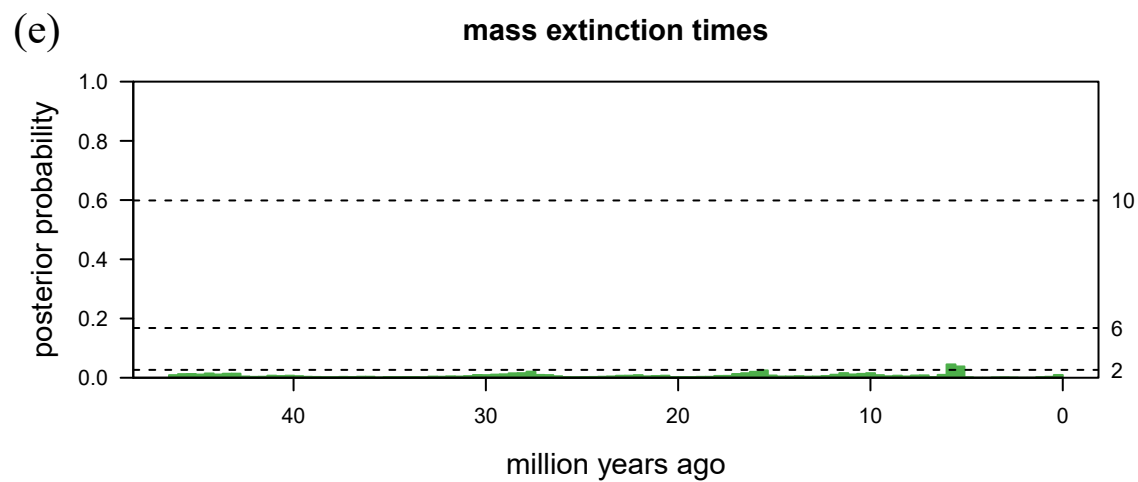

Supplement: Supplementary file 14 — Additional file 14: Fig. S14. Diversification rate inferred from CoMET in TESS. The shaded areas in (a) and (c) indicate 95% confidence intervals of speciation and extinction rates. 2lnBF (the heights of bars) higher than 6 indicate significant speciation rate shift, extinction rate shift, or mass extinction in (b), (d), and (e). [file 12870_2021_3413_MOESM14_ESM.pdf]

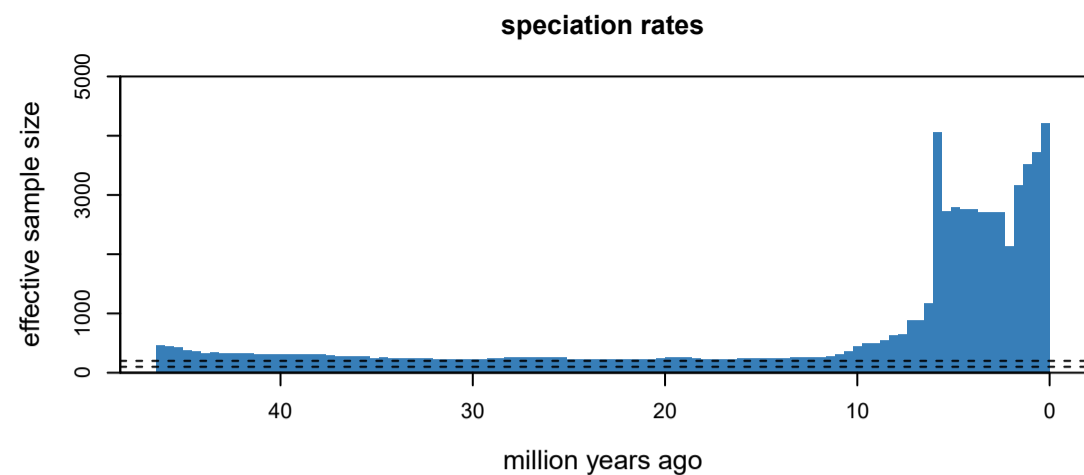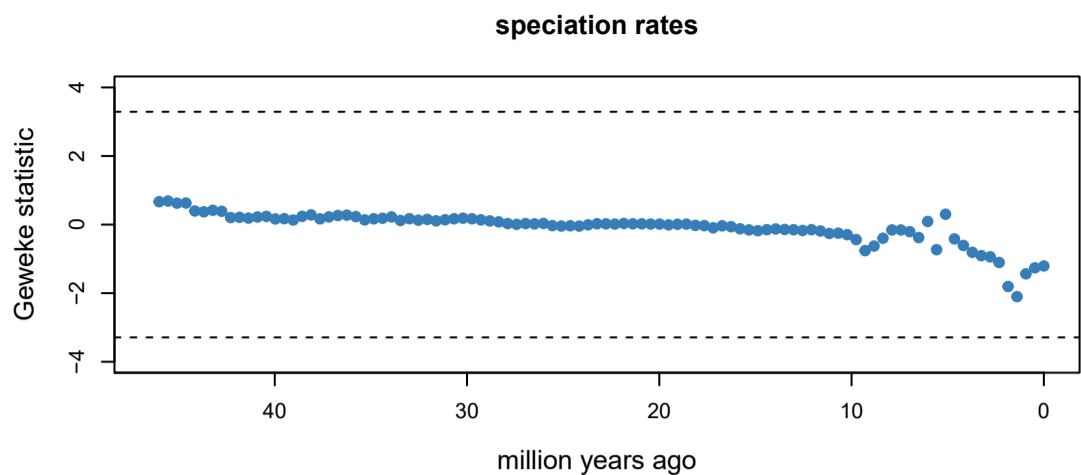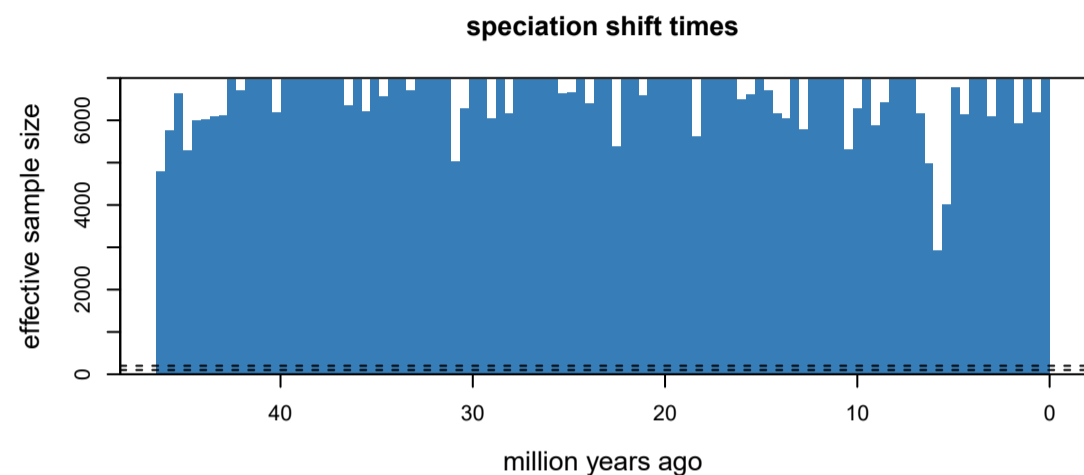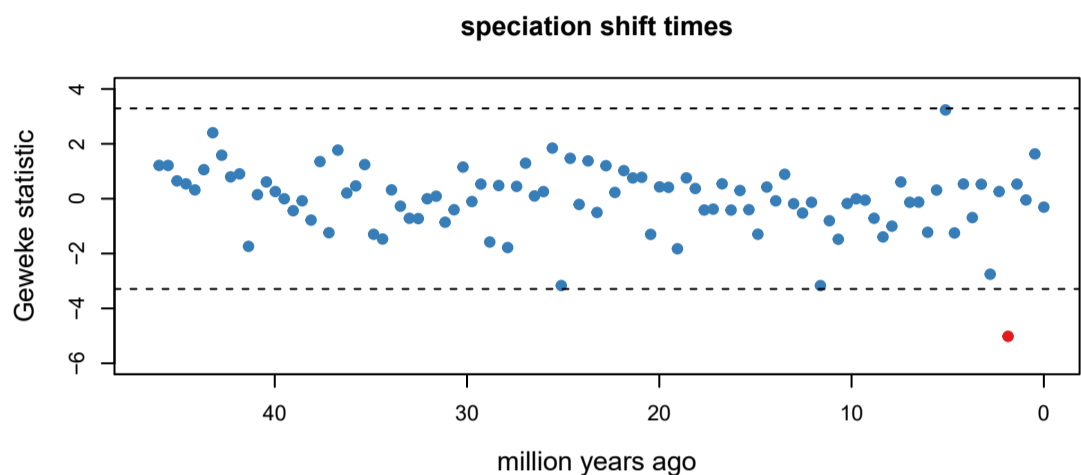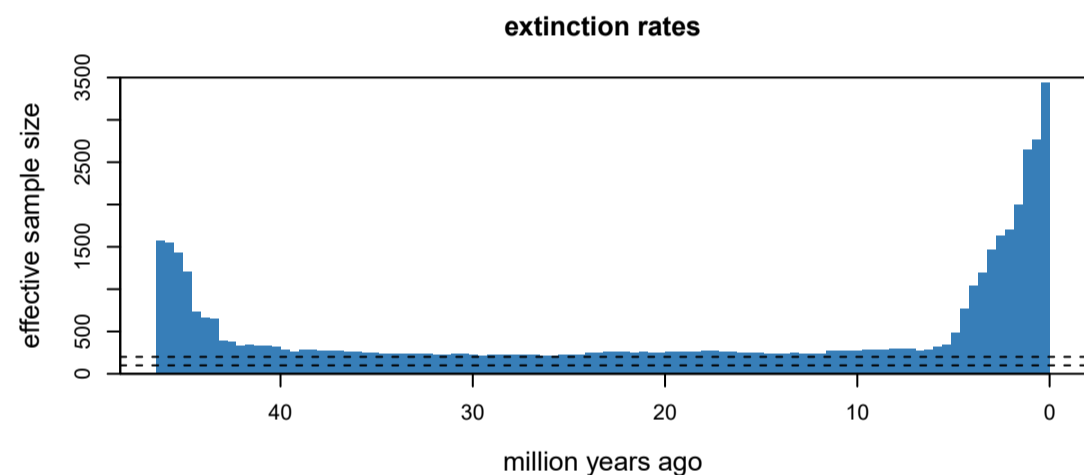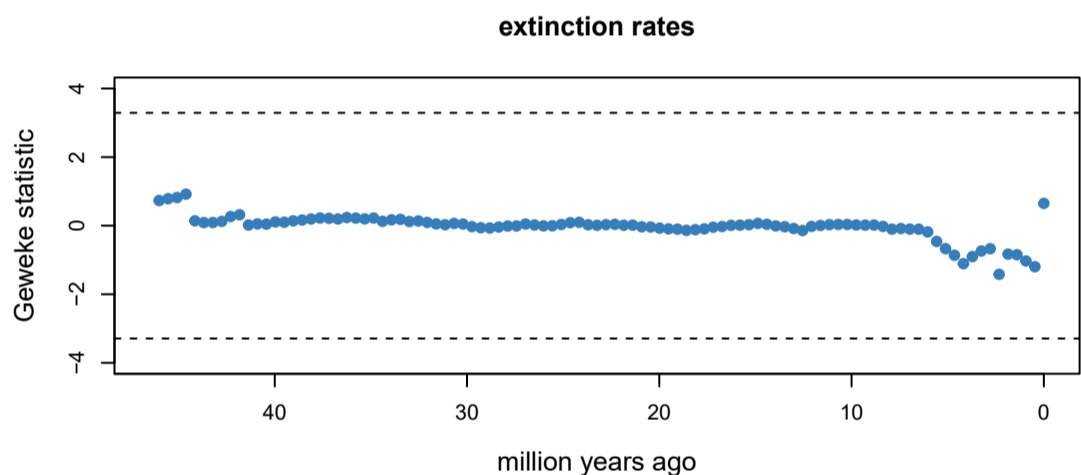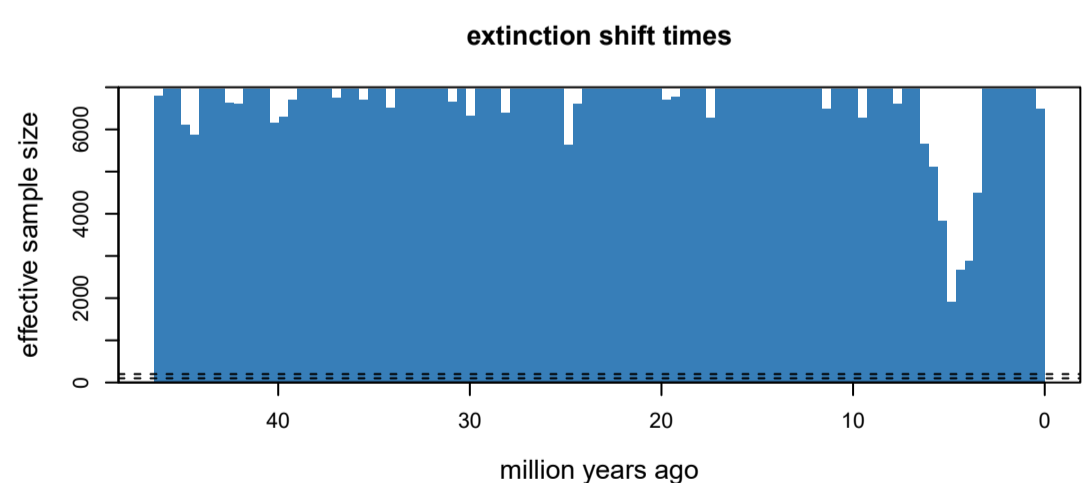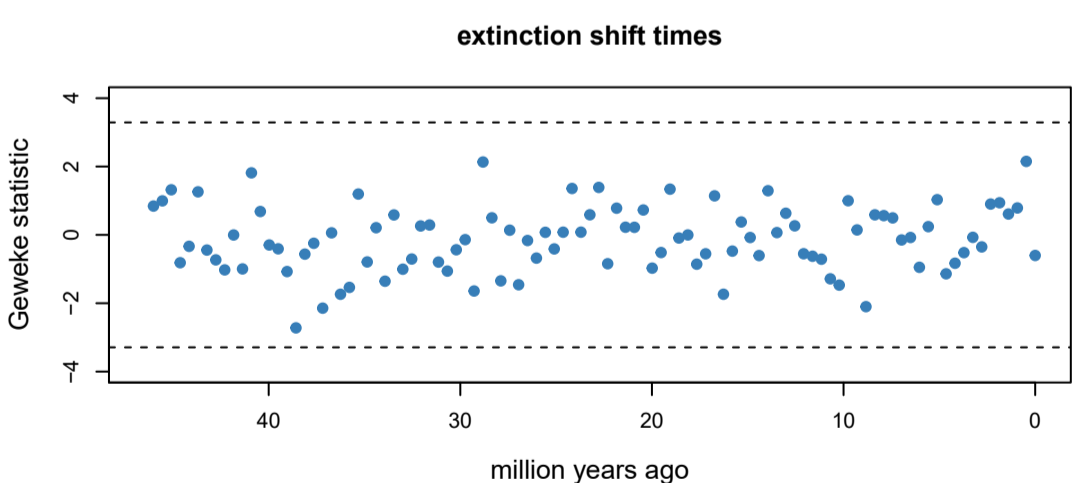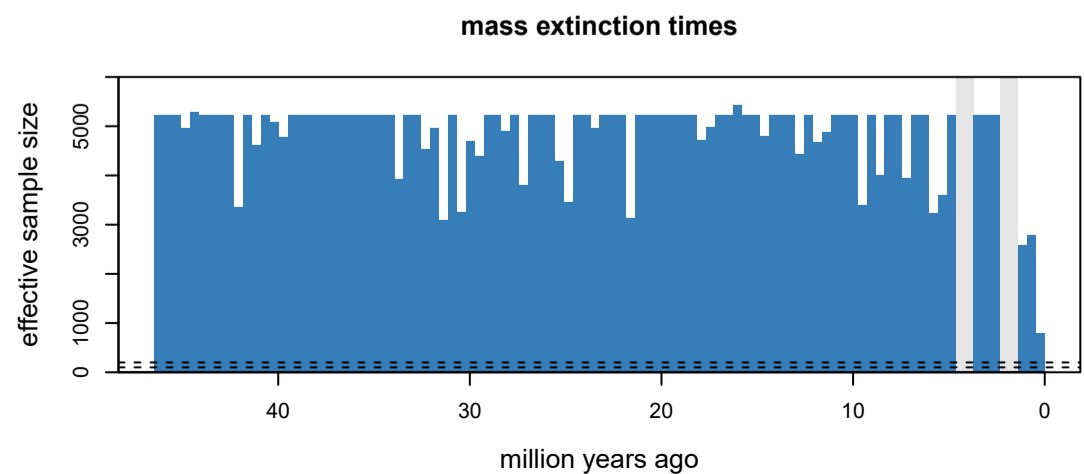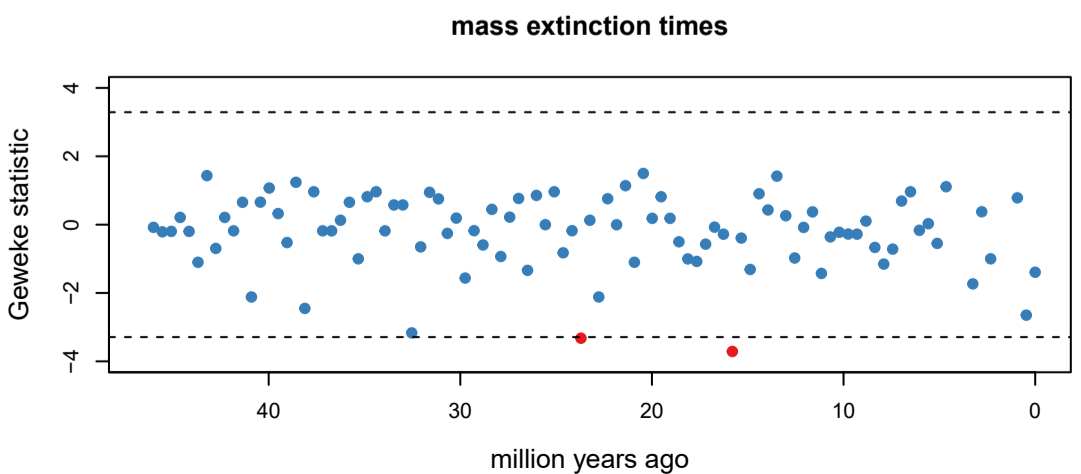

Supplement: Supplementary file 15 — Additional file 15: Fig. S15. The single-chain MCMC diagnostics for a CoMET analysis. The blue bars and dots indicate passed tests, while red bars and dots refer to failed convergence. [file 12870_2021_3413_MOESM15_ESM.pdf]
