# Supplementary material for: Plastid phylogenomics of tribe Perseeae (Lauraceae) yields insights into the evolution of East Asian subtropical evergreen broad-leaved forests
Source: BMC Plant Biol. 2022 Jan 13;22:32. doi: 10.1186/s12870-021-03413-8 (PMC8756638; doi:10.1186/s12870-021-03413-8)
Supplement: Supplementary file 1 — Additional file 1: Fig. S1. Complete plastid genome map of tribe Perseeae. Different genes are color coded. [file 12870_2021_3413_MOESM1_ESM.pdf]

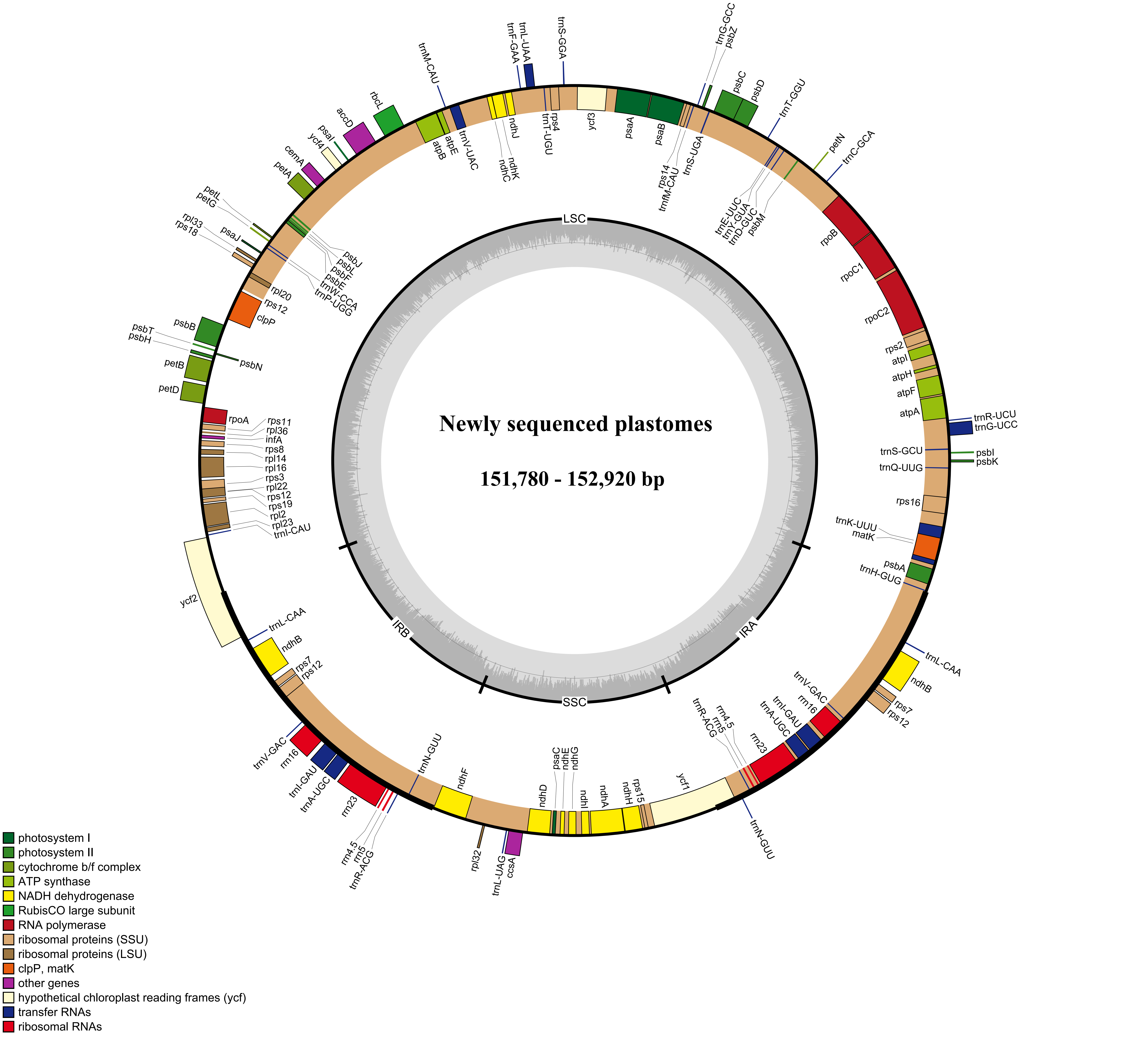

Newly sequenced plastomes
151,780 - 152,920 bp
LSC
IRA
SSC
IRB
trnM-CAU
trnF-GAA
trnL-UAA
trnS-GGA
trnG-GCC
psbZ
psbC
psbD
trnT-GGU
petN
trnC-GCA
rbcL
accD
psaI
ycf4
cemA
petA
atpB
atpE
trnV-UAC
ndhC
ndhK
ndhJ
trnT-UGU
rps4
ycf3
psaA
psaB
rps14
trnfM-CAU
trnS-UGA
trnE-UUC
trnY-GUA
trnD-GUC
psbM
rpoB
rpoC1
rpoC2
rps2
atpI
atpH
atpF
atpA
trnR-UCU
trnG-UCC
trnS-GCU
psbI
psbK
trnQ-UUG
rps16
trnK-UUU
matK
psbA
trnH-GUG
trnL-CAA
ndhB
rps7
rps12
trnV-GAC
rrn16
trnI-GAU
trnA-UGC
rrn23
rrn4.5
rrn5
trnR-ACG
trnN-GUU
ycf1
rps15
ndhH
ndhA
ndhI
ndhG
ndhE
psaC
ndhD
ccsA
trnL-UAG
rpl32
ndhF
petL
petG
rpl33
rps18
psaJ
psbJ
psbL
psbF
psbE
trnW-CCA
trnP-UGG
rpl20
clpP
psbB
psbT
psbH
psbN
petB
petD
rpoA
rps11
rpl36
infA
rps8
rpl14
rpl16
rps3
rpl22
rps19
rpl2
rpl23
trnI-CAU
ycf2
photosystem I
photosystem II
cytochrome b/f complex
ATP synthase
NADH dehydrogenase
RubisCO large subunit
RNA polymerase
ribosomal proteins (SSU)
ribosomal proteins (LSU)
clpP, matK
other genes
hypothetical chloroplast reading frames (ycf)
transfer RNAs
ribosomal RNAs
